# Memory systems modulate crosslinguistic influence on third language morphosyntactic acquisition

**DOI:** 10.1371/journal.pone.0304572

**Published:** 2024-07-11

**Authors:** Emily Shimeng Xu, Stephen Matthews, Virginia Yip, Patrick C. M. Wong

**Affiliations:** 1 Department of Linguistics & Modern Languages, The Chinese University of Hong Kong, Hong Kong SAR, China; 2 Division of Otolaryngology—Head and Neck Surgery, Ann & Robert H. Lurie Children’s Hospital of Chicago, Chicago, Illinois, United States of America; 3 Department of Otolaryngology—Head and Neck Surgery, Feinberg School of Medicine, Northwestern University, Chicago, Illinois, United States of America; 4 Department of Linguistics, The University of Hong Kong, Hong Kong SAR, China; 5 Brain and Mind Institute, The Chinese University of Hong Kong, Hong Kong SAR, China; Basque Center on Cognition Brain and Language, SPAIN

## Abstract

Previous studies on crosslinguistic influence (CLI) on third language (L3) morphosyntactic acquisition have provided support for competing theories about the source(s) of CLI. The present study aimed to test if both L1 and L2 can be the source of CLI, and whether they influence L3 learning in similar or different ways. In particular, we aimed to add to our knowledge of the neural correlates of CLI by conducting an exploratory EEG study to investigate how L1 and L2 CLI affect L3 neural processing. Predictions based on the D/P model, which posited different memory systems sustaining L1 and L2, were tested. The findings confirmed both L1-sourced and L2-sourced facilitation on L3 morphosyntactic acquisition. Specifically, we suggest that L1-similarity showed a consolidating effect on L3 implicit knowledge and neurocognitive internalization, whereas L2-similarity contributed to enhanced L3 metalinguistic knowledge. This preliminary study is the first to investigate the neurocognitive mechanisms underlying CLI in L3 learning by natural language learners.

## Introduction

Learners who speak at least two languages on acquiring a new one exhibit a different acquisition trajectory than their monolingual peers. One factor contributing to the observed differences is the influence of previously acquired languages. Crosslinguistic influence (CLI), also known as language transfer, refers to the phenomenon where knowledge of one linguistic system affects the production or perception of another. Compared to monolinguals whose subsequent language acquisition is potentially influenced only by the native language, bilinguals are potentially affected by both the first and second languages (L1 and L2) when learning a third language (L3). Previous studies on third language acquisition (TLA), particularly those focused on syntax, have collected substantial yet conflicting findings on the source(s) of CLI and its ramifications. Considering the dynamic interactions among L1, L2, and L3, the crosslinguistic influence triggered by overlaps between L1 or L2 and L3 is far from being well understood, and the effect of such influence has not been extensively investigated using neurocognitive methods. Nonetheless, previous findings on the overlapping neurocognitive systems involved in L1 and L2 processing [1] and the different memory systems for L1 and L2 storage [2] allow us to make predictions about whether L1 or L2, or both, can be the source of CLI and about the corresponding influence of L1 or L2 on the acquisition of L3.

In this study, we aimed to explore the transferability of L1 and L2 by testing bilingual adults’ performance on L3 morphosyntactic features that overlap with either L1 or L2 and demonstrating how L1 and L2 transfer influence L3 learning differently. Additionally, most empirical studies on CLI have adopted behavioral measures to investigate L1 and L2 influence on L3 perception or production [3–6], leaving the underlying neurocognitive mechanisms of the observed CLI undetermined. To shed light on the neural operations underlying CLI, we coupled behavioral experiments with a neuroimaging component, namely electroencephalography (EEG) to provide evidence of learners’ brain activities that arise through crosslinguistic interaction. Furthermore, existing neurocognitive investigations of L3 morphosyntactic CLI mostly adopted artificial language training, and hence have limited generalizability to real-world language learning. The present exploratory study is the first to investigate the neural underpinnings of CLI in third language acquisition with authentic natural language learners.

## Background

### Behavioral evidence for similarity-driven crosslinguistic influence

Studies on morphosyntactic crosslinguistic influence (CLI) have yielded mixed findings regarding whether L1 or L2 is the source of CLI in L3 acquisition and how L1- or L2-sourced CLI affects L3 learning, giving rise to divergent theories of CLI in L3 (morpho)syntactic acquisition. The Cumulative Enhancement Model (CEM) [7] builds upon findings of additive effects of L1 and L2 on L3 acquisition induced by L1-L3 or L2-L3 feature-specific similarity for a syntactic structure [7,8]. The Linguistic Proximity Model (LPM) [9,10] similarly recognises cumulative feature-specific CLI from L1 and L2 but acknowledges its possible hindrance on L3 learning resulting from wrongly perceived similarity between L1/L2 and L3. Other theories see either L1 or L2 as the main source of CLI. The L1-dominant hypothesis [11] predicts the native language to be the privileged transfer source and argues for overgeneralization of L1 syntactic rules to L3. The L2-Status Factor model [12–14] sees the L2 as the primary source of syntactic CLI. The Typological Primacy Model (TPM) [15], on the other hand, suggests a critical role for psychotypological similarity between the L1/L2 and L3 in determining CLI source [16]. The TPM speculates that once a source language is selected, its entire set of syntactic properties is generalized to the L3, resulting in potentially erroneous L3 perception and production. Moreover, studies on L1/L2 crosslinguistic influence on L3 predominantly examined sentence-level syntactic properties (for word order, see [10,17]; for the null-subject parameter, see [18]; for negation placement, see [12]; for relative clauses, see [7]), leaving word-level ones (such as morphosyntactic inflections) largely unaddressed.

Among studies that have investigated morphosyntactic CLI, Diaubalick et al. [19] tested the facilitative effect of L2 morphosyntactic knowledge on the acquisition of L3 telic predicate inflection, a feature existing in Romance languages but lacking in Germanic languages. L1-German learners of L3-Spanish with varying proficiency in another L2 Romance language were tested. Findings revealed that learners’ L3 performance was positively correlated to their proficiency in the L2 Romance language. Another study [20] examined L2-English learners of L3 French with two different L1s: Spanish and Turkish, which respectively resemble and differ from French. Overall, the L1-Spanish group performed comparably to French natives and significantly better than the L1-Turkish group. Additionally, the L1-Turkish group’s performance on French article assignment, a feature shared by English and French, positively correlated to their L2-English proficiency. In sum, existing empirical findings from behavioral tasks provided evidence for both L1 and L2 being possible sources of positive CLI on L3 acquisition in cases of L1-L3 and L2-L3 congruency. However, the critical question of how L1-sourced and L2-sourced CLI differ in their effects on L3 processing remains unanswered.

One prevalent theory to account for the processing difference between L1 and L2 is the Declarative/Procedural Model (D/P model) [2,21]. The fundamental hypothesis of the D/P model is that the declarative and procedural memory systems subserve different functions in the processing, storage, and use of L1 and L2. In terms of (morpho)syntax, procedural memory is engaged in acquiring and sustaining L1 structural regularities which are stored as implicit knowledge with spontaneous and automatic access, whereas declarative memory sustains subsequently acquired grammatical systems as explicit knowledge with conscious and intentional access, especially if the grammar was acquired in a classroom setting where explicit knowledge was emphasized. The L2 status factor model [13] previously drew upon the D/P model in support of the hypothesized transfer priority of L2 syntactic properties. However, it failed to elaborate on the situation where L1 is the only transferable source, i.e., the only overlapping language with L3 for a specific feature. As predicted by the D/P model, under the condition where L2 exposure and utility are limited, L1 and L2 have different storage and access settings and consequently have different influences on the acquisition of a sequential language. Specifically, with L1 stored and accessed implicitly and L2 explicitly, it is possible that L1, when shares structural similarity with L3, promotes the development of implicit L3 knowledge whereas L2 facilitates the processing of explicit L3 knowledge. Researchers have examined implicit and explicit (morpho)syntactic knowledge via factor-analytic methods and found that tasks directing learners’ attention to the structural integrity of L2 primarily target explicit grammatical knowledge, while those distracting learners from structural information tap into implicit grammar knowledge [22]. The self-paced reading task (SPRT) and the untimed grammaticality judgement task (GJT) [23,24] are commonly used to test L2 learners’ implicit and explicit knowledge respectively. The judgement accuracy of untimed GJT reflects explicit morphosyntactic knowledge, whereas the reading time for the word of interest during SPRT indexes implicit knowledge [25]. Previous studies have applied these two tasks to examine learners’ implicit and explicit processing of L2 grammar under the influence of L1-L2 structural similarity. Findings revealed that, while learners displayed comparable explicit knowledge of L1-similar and L2-unique structures, they exhibited implicit knowledge only of L2 structures with similarity to L1 [26,27], indicating that L1-L2 shared structures were accessed implicitly via more automatic processing possibly supported by the procedural memory, while others were accessed explicitly in the declarative memory. Generalizing such engagement of declarative and procedural memories to L3 morphosyntactic processing, we predicted that L1-L3 similarity would be conducive to the development of implicit L3 morphosyntactic knowledge, while L2-L3 similarity would enhance explicit L3 knowledge.

### Neuroimaging evidence for similarity-driven crosslinguistic influence

Previous studies that applied neuroimaging techniques to examine learners’ processing of newly acquired morphosyntactic structure were focused on second language acquisition (SLA). EEG findings on language processing have identified event-related potentials (ERPs) that are critical to language processing: a left anterior negativity (LAN) that peaks at around 250 to 300-millisecond (ms) post-stimulus onset, a positive deflection that peaks at roughly 600ms after stimulus presentation (i.e., the P600) and a negative deflection with a post-stimulus-onset latency of 400 to 500ms (i.e., the N400) [28–32]. Studies on L1 processing found that morphosyntactic oddity commonly elicited a LAN or an early left anterior negativity (ELAN, an early version of the LAN component that peaks at roughly 100ms post-stimulus onset) followed by a P600 response whereas semantic improbability regularly induced an N400 component (for a review, see [33]), and such observation was consistent across languages [34–39]. EEG studies on L2 morphosyntactic processing revealed that early stage L2 learners typically show either a meaning-oriented N400 or no robust brain reactions towards L2 morphosyntactic violations. As proficiency improves, learners’ responses to grammatical anomaly gradually shift to the form-sensitive P600, occasionally preceded by LAN, which resembles the syntactic processing of native speakers [40,41]. However, contrary to the typical observation, early-stage L2 learners were found to exhibit a native-like P600 response when processing violations of a L2 structure formed similarly in their L1, suggesting early proceduralization of the L1-shared L2 features [42–45].

In TLA, empirical studies using neural methodologies are limited. One recent EEG study [46] on morphological L1/L2 CLI on L3 adopted an artificial language learning paradigm and trained L1-Spanish L2-English speakers with either mini-English or mini-Spanish. Both artificial languages (ALs) had the gender feature that exists in Spanish but not in English. Although behavioral outcomes revealed no difference between the two AL groups, the mini-English group showed a right-lateralized early anterior negativity brain response to gender violation, whereas the violation in mini-Spanish elicited a widely distributed early positivity. Although the findings do not conclusively corroborate the effect of the crosslinguistic influence from either L1 or L2, they indicate differences in the neurocognitive processing as a result of L1- or L2-similarity.

## The current study

In the current study, a web-based behavioral experiment and an EEG experiment were conducted to explore the effects of L1 and L2 crosslinguistic influence (CLI) on L3 morphosyntactic acquisition, the respective neurocognitive mechanisms of L1 and L2 CLI on L3 processing and the role of memory systems in influencing L1 and L2 CLI.

L3 Korean learners with Cantonese as L1 and English as L2 were recruited and tested on three Korean morphosyntactic features: the prenominal adjectival marker, simple past-tense verb inflection and nominative-accusative case marking. The prenominal adjectival marker or attributive marker is a morphosyntactic feature instantiated similarly in the learner’s L3 Korean (1a) and L1 Cantonese (1b) but absent from L2 English. Prenominal adjectives that function as modifiers within noun phrases (NPs) are formed regularly with the prenominal adjective morpheme (PreN), i.e., the attributive marker -n, in Korean and with ge3 in Cantonese [47,48], but are not marked morphosyntactically in English. Past-tense verb inflection, lacking in Cantonese, exists in both L3 Korean (2a) and L2 English (2b) with similar morphological form. Simple past tense (PAST) verbs are regularly inflected with the -ss suffix in Korean, which is structurally and functionally similar to the regular -ed inflection of English verbs. Unlike Cantonese or English, Korean uniquely marks the object of a verb phrase with the accusative case (ACC), instantiated as a suffix -eul attached to the object noun (see 3). Although the surface forms of all three morphosyntactic structures vary based on the ending sound of the root adjective/verb/noun, the suffixes listed above and bolded in the example sentences are mandatory and regularly present in their respective structures.

**Table:** 

1a	Korean	yeppeu-**n** yeoja	pretty woman
		pretty-PreN woman	
		
1b	Cantonese	piu1loeng6-**ge3** neoi5zai2*	
		pretty-PreN woman	
* numbers indicate Cantonese tones			
2a	Korean	yeonghwa-reul bwa-**ss**-da.	watched movie
		movie-ACC watch-PAST-DECL*	
		
2b	English	watch**-ed** movie	
		watch-PAST movie	
* DECL: declarative mood			
3	Korean	chaeg**-eul** ilg-neun-da	read a book
		book-ACC read-INDI*-DECL	
* INDI: indicative mood			

We hypothesized that 1) similarity-driven CLI from both L1 and L2 would enhance the learner’s explicit metalinguistic awareness of the relevant L3 morphosyntactic structures, but 2) only L1-sourced CLI would facilitate the proceduralization of L3 morphosyntax, thus contributing to 3) native-like neurocognitive processing of the L3 structure shared with L1.

## Experiment 1: Web-based behavioral experiment

The behavioral experiment was built and conducted using the online experiment builder Gorilla (www.gorilla.sc, see [49] for detailed description; for validation of the precision and accuracy of Gorilla, see [50,51]). Learners’ implicit and explicit L3 morphosyntactic knowledge was tested respectively with a self-paced reading task (SPRT) and an untimed grammaticality judgement task (GJT) to reveal the different effects of L1- and L2-sourced CLI on L3 morphosyntactic acquisition.

### Method

Both Experiment 1 and Experiment 2 were carried out in accordance with the ethical standards of the institutional research committee of the Chinese University of Hong Kong. The protocols were approved by the Joint Chinese University of Hong Kong—New Territories East Cluster Clinical Research Ethics Committee. All subjects in Experiment 1 and Experiment 2 gave written informed consent in accordance with the Declaration of Helsinki. Recruitment of experiment participants started on October 1^st^, 2020, and ended on October 31^st^, 2021.

#### Participants

Fifty-three learners of Korean between ages 18 to 25 (mean = 20.38, SD = 0.94) were recruited for the online experiment, along with a control group of 26 Korean native speakers aged 18 to 30 (mean = 23.23, SD = 2.72). Learners were native speakers of Cantonese, had learned English as the L2 from the age of 2 to 6 years old, and were L3 Korean learners enrolled in Korean courses offered by a university in Hong Kong. 27 of the 53 learners were enrolled in advanced-level Korean courses at the time of testing (i.e., the high-proficiency group) and the other 26 were enrolled in beginning or intermediate-beginning courses (i.e., the low-proficiency group). Class assignment was determined by students’ performance in a comprehensive evaluation of Korean listening, vocabulary, reading, writing, and speaking skills created by Korean native language teachers for each level of the Korean course. Learners were only allowed to register for a higher level of the Korean course if they have passed the evaluation of the preceding level. All learners received formal Korean instruction solely from the courses offered by the program. It is worth noting that, despite the participants’ young age of English acquisition, we do not consider their English proficiency native-like with implicit access, especially so for grammar. Hong Kong local students commonly learn English in a rule-based, classroom-setting context with English exposure typically limited to receiving lectures and doing coursework. The majority of Hong Kong young adults, like those tested in our study, use solely Cantonese for communicating with peers and parents and for conducting daily life routines. To guarantee that the learner group indeed learned English explicitly, we specifically screened their background information and made sure that all of the subjects received mainstream English education in non-international schools. According to the Declarative/Procedural Model (D/P model) [2], L2 grammar acquired via a classroom-setting instruction method is typically stored as explicit knowledge in the declarative memory system. Furthermore, previous empirical study found that explicit, rule-based language learning resulted in a processing outcome in high-proficiency L2 learners that was dissimilar to the native speakers, supporting that explicitly acquired L2 grammar might not be proceduralized into implicit knowledge in L2 learners [28]. As our tested L2-sourced grammar did not overlap with or exist in the L1-Cantonese and our learners acquired English in an explicit teaching context with a limited amount of English exposure in their daily communication, we believe that our learners’ L2-English grammar was stored as explicit knowledge in the declarative memory system.

#### Materials

The behavioral experiment tested three Korean morphosyntactic structures: 1) prenominal adjective marker, 2) past-tense inflection and 3) nominative-accusative case marking, respectively corresponding to the three conditions of ADJ, Verb and Case. 340 sentences were generated, with 80 experimental sentences for each condition and 100 fillers. Ungrammatical sentences were formed with relevant morphosyntactic violations. Specifically, for the ADJ condition, the ungrammatical stimulus was formed by replacing the prenominal adjectival marker with the postnominal one -o; for the Verb condition, the inflectional violation was generated by using present tense verbs when past tense was required; for the Case condition, the accusative marker -eul of the objectives was replaced with the nominative markers as case marking violation. Table 1 shows sample stimuli and violation manipulation. Each stimulus sentence contained a subject followed by four content words, with word frequency (freq.) within each category (i.e., location, object, verb) controlled across structural conditions (Table 2). The frequency data were extracted from the Modern Korean Usage Frequency Survey [52] published by the National Institute of Korean Language of the Republic of Korea.

**Table 1:** Example sentences for each condition.

**ADJ condition (L1-L3 congruent)**					
Grammatical example:	재민씨-는	집-에서	**매운**	비빔밥-을	먹어요.
	Jaeminssi-neun	jip-eseo	maeu-**n**	bibimbap-eul	meogeoyo.
	Jaemin-TOP	home-LOC	spicy-**PreN**	bibimbap-ACC	eat.
Ungrammatical example:	재민씨-는	집-에서	**매워**	비빔밥-을	먹어요.
	Jaeminssi-neun	jip-eseo	maeu-**o**	bibimbap-eul	meogeoyo.
	Jaemin-TOP	home-LOC	spicy-**PostN**	bibimbap-ACC	eat.
**Verb condition (L2-L3 congruent)**					
Grammatical example:	애니씨-는	어제	저녁후-에	영화-를	**봤어요.**
	Aenissi-neun	eoje	jeonyeokhu-e	yeonghwa-reul	bwa**sseo**yo.
	Annie-TOP	yesterday	after dinner-LOC	movie-ACC	watch**ed**.
Ungrammatical example:	애니씨-는	어제	저녁후-에	영화-를	**봐요.**
	Aenissi-neun	eoje	jeonyeokhu-e	yeonghwa-reul	bwayo.
	Annie-TOP	yesterday	after dinner-LOC	movie-ACC	watch.
**Case condition (L3-unique)**					
Grammatical example:	마틴씨-는	아침-에	테니스-**를**	항상	쳐요.
	Matinssi-neun	achim-e	teniseu-**reul**	hangsang	chyeoyo.
	Martin-TOP	morning-LOC	tennis-**ACC**	often	play.
Ungrammatical example:	마틴씨-는	아침-에	테니스-**가**	항상	쳐요.
	Matinssi-neun	achim-e	teniseu-**ga**	hangsang	chyeoyo.
	Martin-TOP	morning-LOC	tennis-**NOM**	often	play.

Grammaticality manipulations are in bold font; critical words in the self-paced reading analysis are underlined. TOP: Topic marker; LOC: Location marker; PreN: Prenominal adjective marker; PostN: Postnominal adjective marker; ACC: Accusative case marker; NOM: Nominative case marker.

**Table 2:** Mean (SD) word frequency of each category. Word frequency is calculated as the number of occurrences out of 3-million-word entries.

	Location (noun) freq.	Object (noun) freq.	Verb freq.
**ADJ**	525.54 (650.28)	553.30 (531.30)	4970.21 (9373.12)
**Verb**	554.64 (590.53)	559.28 (638.83)	4707.86 (9335.65)
**Case**	542.60 (520.78)	513.71 (990.93)	4951.24 (10265.19)

#### Procedure

Prior to starting the experimental task, each participant filled out an online questionnaire that collected background information, shown in Table 3, including their ages of acquisition of L2 (i.e., English) and L3 (i.e., Korean) and the time they spent practicing Korean outside the classroom, excluding watching Korean drama, listening to Korean songs or other kinds of entertainment.

**Table 3:** Learners background information.

	Age	AoA_L2	AoA_L3	Out-class L3 (in hours)
**High proficiency**	20.64 (0.99)	4.46 (1.82)	15.42 (2.45)	1.25 (0.61)
**Low proficiency**	20.13 (0.85)	5.3 (1.22)	15.43 (2.64)	1.17 (0.35)

L2: English; L3: Korean; AoA: Age of Acquisition, calculated in years. Out-class L3: Hours spent practicing Korean outside the classroom. All are mean (SD) values.

All participants then undertook both tasks in the order of SPRT followed by GJT, to avoid concentration on grammaticality during the SPRT. Each task had 4 versions with stimulus grammaticality and response hands counterbalanced.

**Fig 1:**
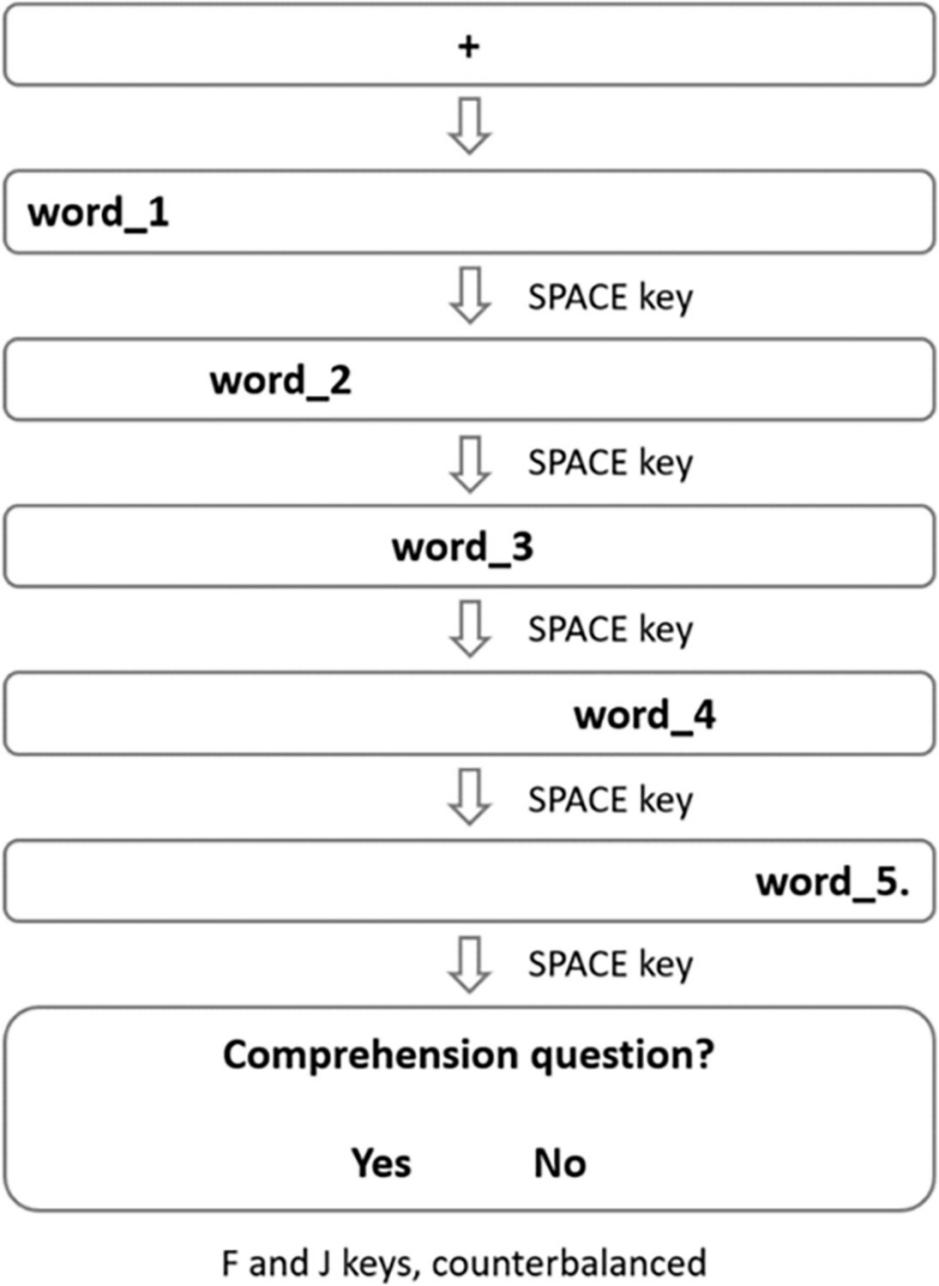
Trial demonstration of the SPRT.

*Self-paced reading task*. The SPRT included 40 experimental sentences for each condition with half containing morphosyntactic violations, half conforming to grammaticality, and 30 fillers, resulting in a total of 150 stimuli presented in randomized order. Participants were instructed to read a sentence word by word as quickly as possible. For each sentence, the first word appeared on the leftmost side of the screen, with the following words triggered by each pressing of the space bar (i.e., the moving window paradigm, see [23]), with the final word followed by a period to indicate the sentence end (Fig 1). Each sentence was followed by a comprehension question requiring a yes/no response.

**Fig 2:**
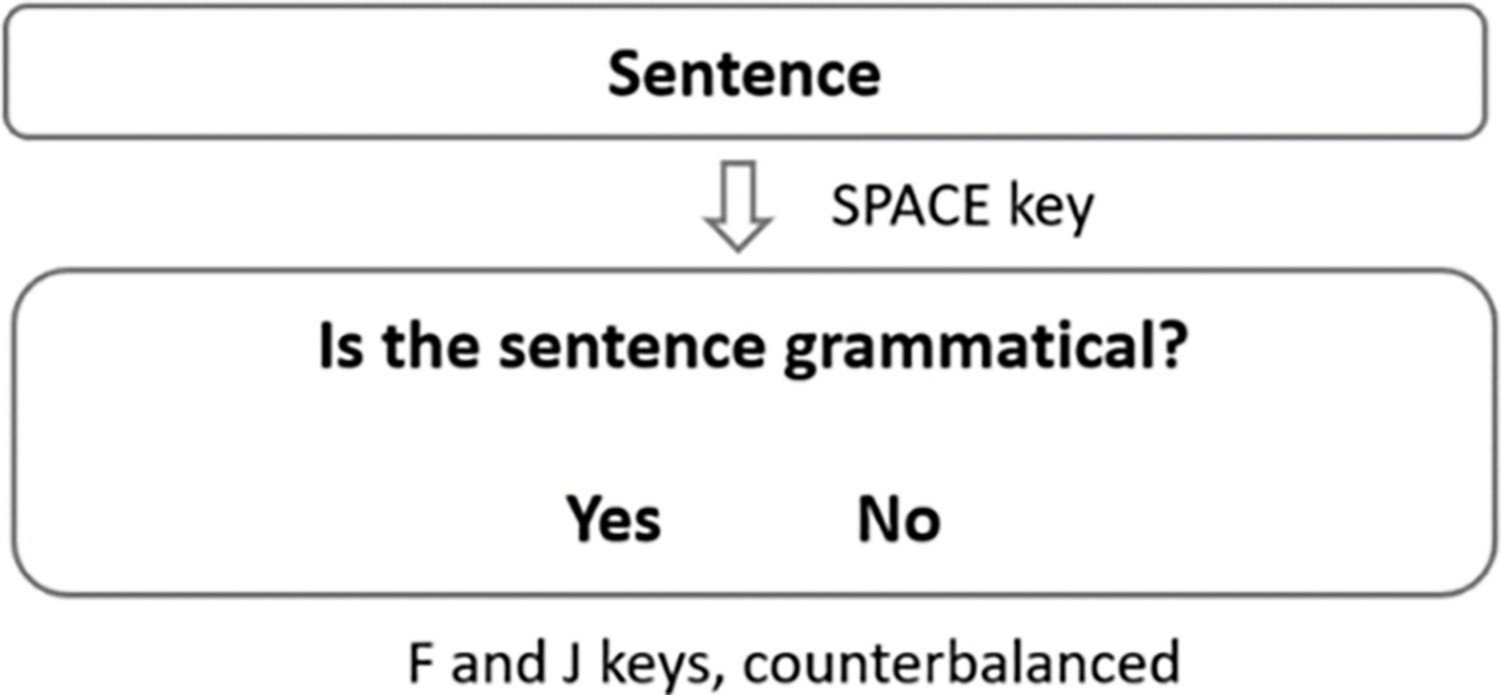
Trial demonstration of the untimed GJT.

*Untimed grammaticality judgement task*. The untimed GJT contained 180 stimulus sentences: 20 grammatical and 20 ungrammatical sentences for each structure and 60 fillers, presented in randomized order. Participants were instructed to read each sentence for as long as needed and then press the space bar to proceed to the judgement page that prompted them to make a binary yes/no response (Fig 2).

#### Data preparation

For the untimed GJT, accuracy score was calculated as the ratio of correctly judged sentences over all sentences responded to for each structural condition. Participants with an accuracy score below 0.5 for any of the three conditions were rejected from data analysis, resulting in the rejection of 1 high proficiency and 2 low proficiency learners.

**Fig 3:**
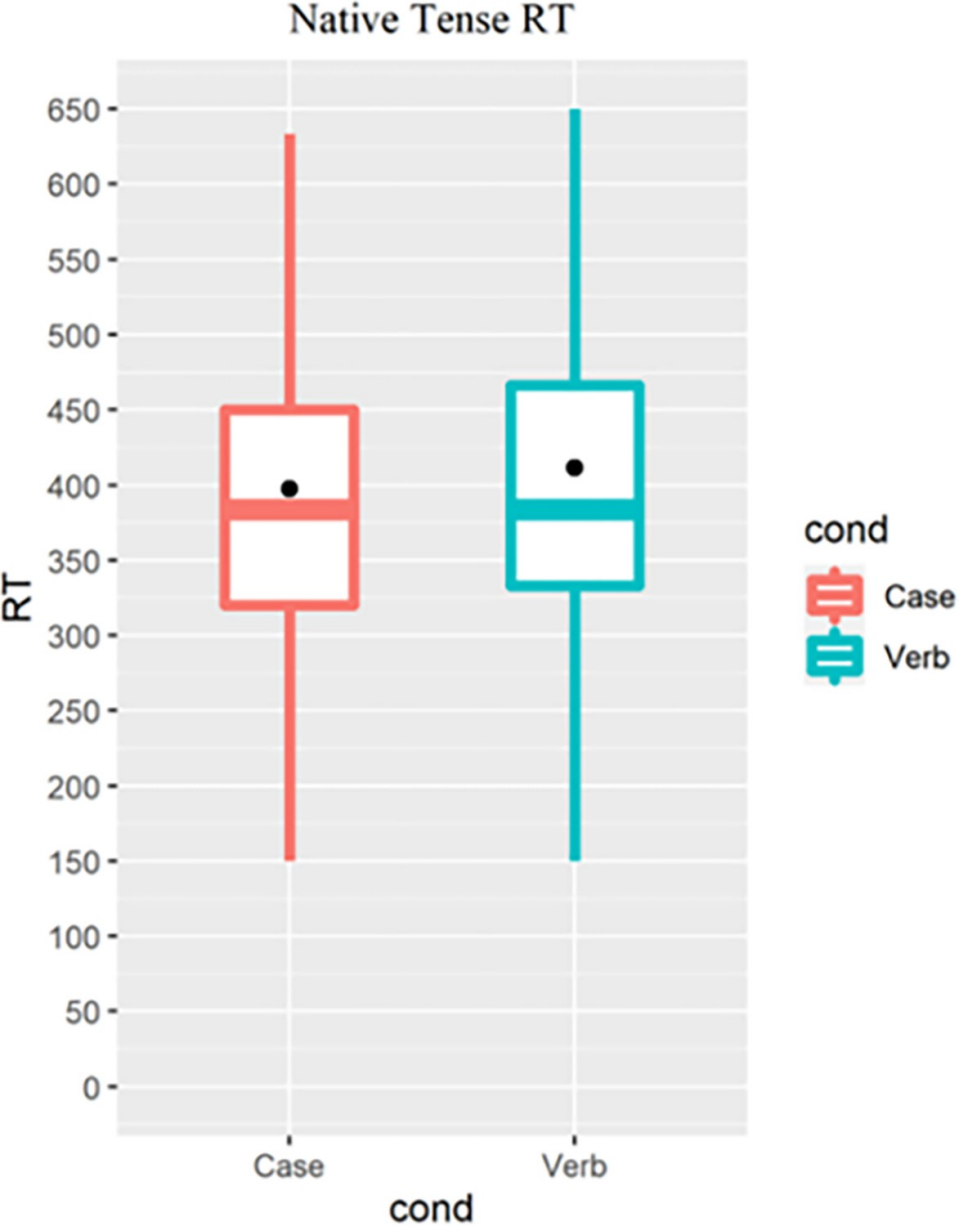
Native Koreans’ RT for the past-tense verbs (Verb) and for the present verbs (Case).

For the SPRT, statistical analysis was conducted on the reaction time (or reading time, RT) for the conditional critical word, as highlighted in Table 1. The critical word was the region where syntactic violation can be determined. The regions of interest (i.e., the critical words) were confirmed by two Korean natives who were asked to read all the experimental sentences word by word and rate each concatenated word/phrase until they reached a confident grammaticality judgement for the sentence. Only participants with at least 50% accuracy for the SPRT comprehension questions were included, which resulted in no further participant rejection; trials with RT under 150 milliseconds(ms) or out of the conditional ± 2.5SD range were rejected. For the Verb condition, as evidenced by the examples in Table 3.1, grammatical verb forms were consistently longer and more morphologically complex than the ungrammatical verb form. Earlier literature [53–55] has revealed a word length effect during sentence reading, which was observable in our native speakers’ longer RT for past-tense verbs than present-tense verbs in grammatical sentences (Fig 3). To minimize such effect, native speakers’ reading times for present- and past-tense verbs were fitted into a linear mixed model to estimate the effect of word length. The estimated effect was then regressed from all the raw reading time of the past tense verbs, following Lago et al. [54]. Native speakers’ reading time is expected to show a consistent and less variable effect of the word length and hence was used to estimate the effect.

Statistical analysis was conducted with the post-rejection, corrected RT data from 25 high proficiency learners, 24 low proficiency learners and 26 native Koreans.

### Statistical analysis and results

Statistical analysis was carried out in R (Version 4.1.0, R Core Team, 2021).

#### Grammaticality judgement accuracy

**Fig 4:**
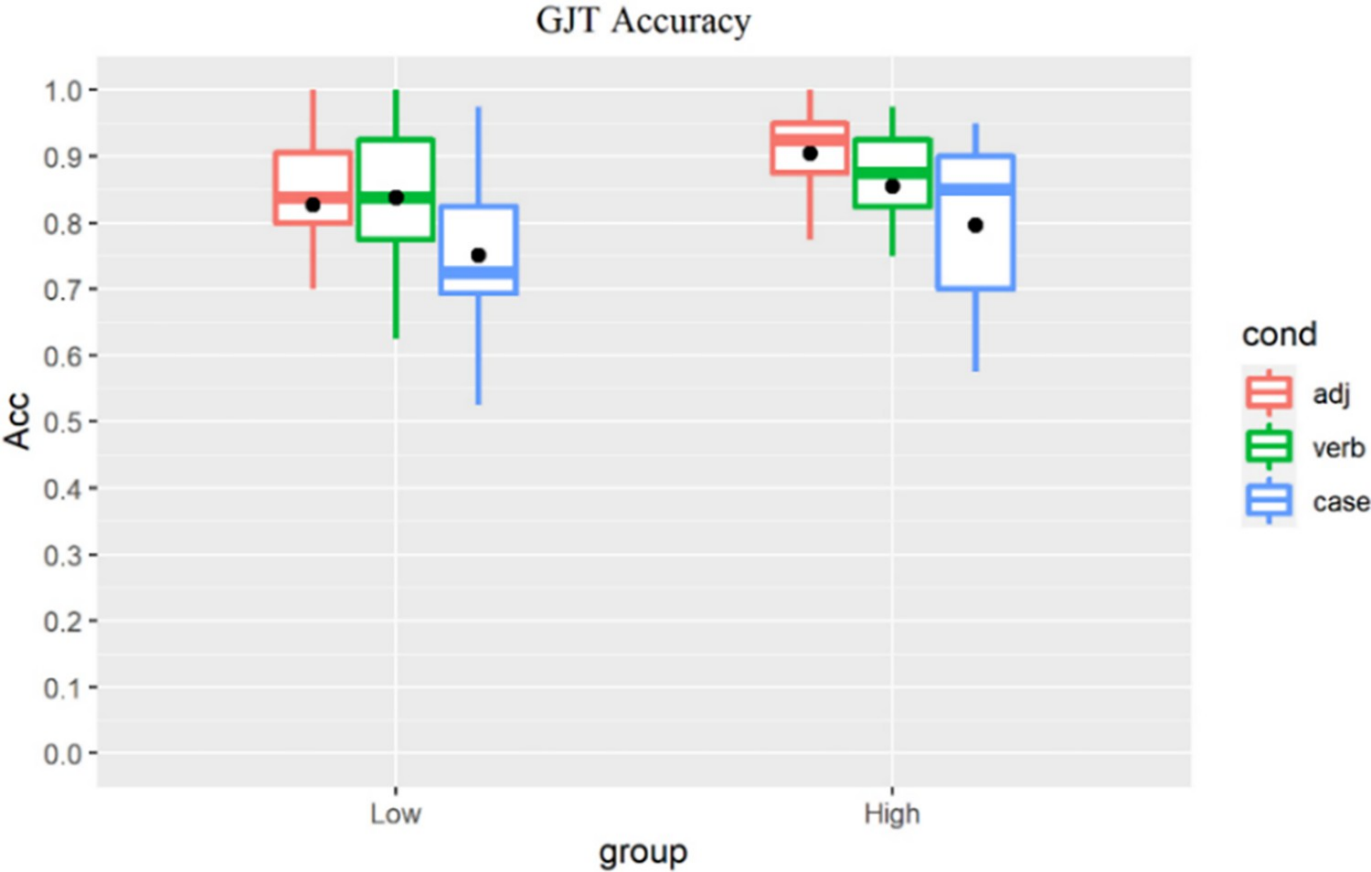
All learners’ GJT results by group (dot indicates the group-level mean).

As Shapiro-Wilk tests revealed that learners’ accuracy scores (Table 4 and Fig 4) were not normally distributed, nonparametric tests were used.

**Table 4:** Group-level GJT accuracy means and SDs.

	ADJ	Verb	Case
**Low**	0.83 (SD = 0.12)	0.84 (SD = 0.10)	0.75 (SD = 0.11)
**High**	0.91 (SD = 0.07)	0.86 (SD = 0.12)	0.80 (SD = 0.12)
**Native**	0.95 (SD = 0.06)	0.97 (SD = 0.02)	0.93 (SD = 0.04)

**Fig 5:**
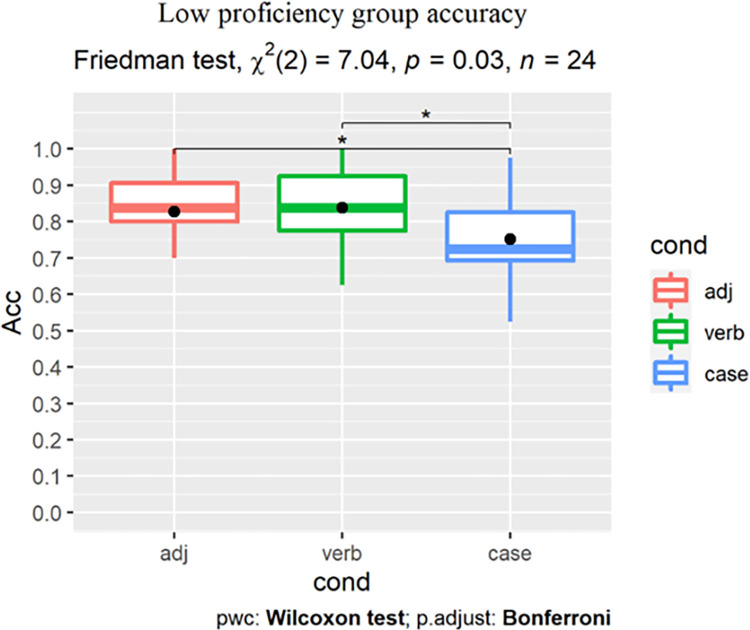
Low proficiency learners’ accuracy (%).

To reveal the main effect of condition, we conducted within-group Friedman tests with post-hoc pairwise Wilcoxon signed-rank tests with Bonferroni correction, reported along with the effect sizes (Pearson’s r). For the low proficiency group, analysis revealed a main effect of condition (χ^2^(2) = 7.04, p = 0.03), with significantly higher scores in the L1-shared ADJ (adjusted p = 0.017, r = 0.57) and the L2-shared Verb conditions (adjusted p = 0.034, r = 0.52) than in the L3-unique Case condition. Accuracy for the ADJ and Verb conditions did not differ robustly (Fig 5).

**Fig 6:**
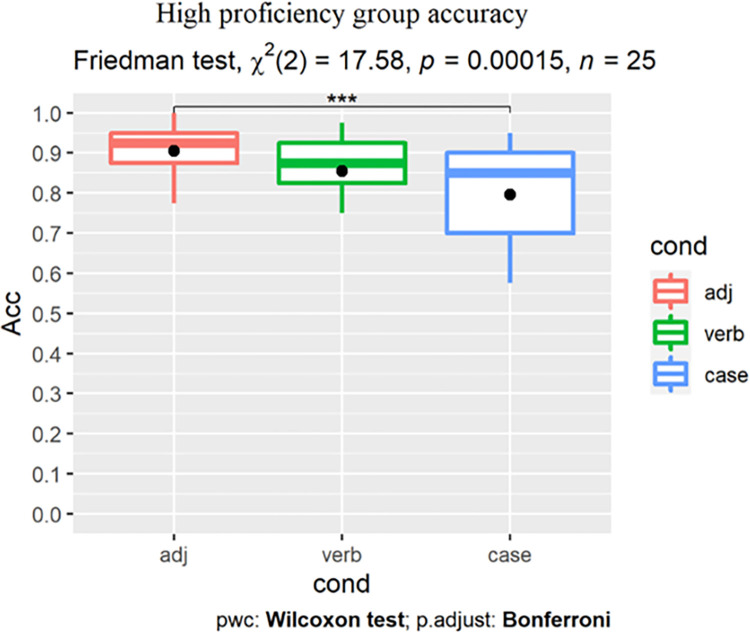
High proficiency learners’ accuracy (%).

For the high proficiency group, the Friedman test returned a main effect of condition (χ^2^(2) = 17.58, p<0.001). Post-hoc tests revealed that learners showed the highest accuracy for the ADJ condition, which was robustly higher than the Case condition (adjusted p<0. 001, r = 0.846). Their scores for the Verb condition were lower than for the ADJ condition (adjusted p = 0.22, r = 0.369) but higher than for Case (adjusted p = 0.21, r = 0.364), yet neither difference reached statistical significance (Fig 6).

**Fig 7:**
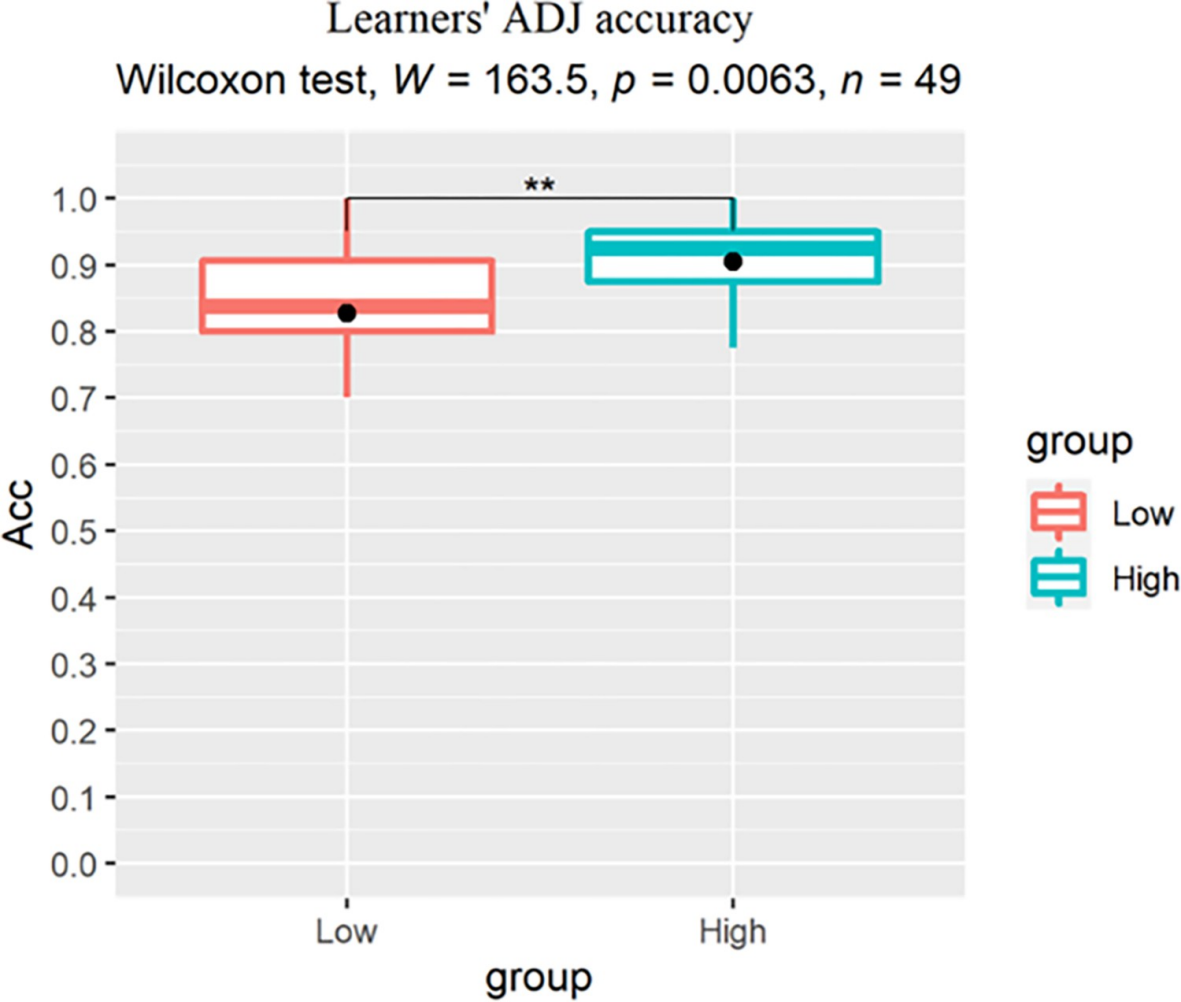
Learners’ accuracy (%) for the ADJ structure, dots indicate group-level means.

**Fig 8:**
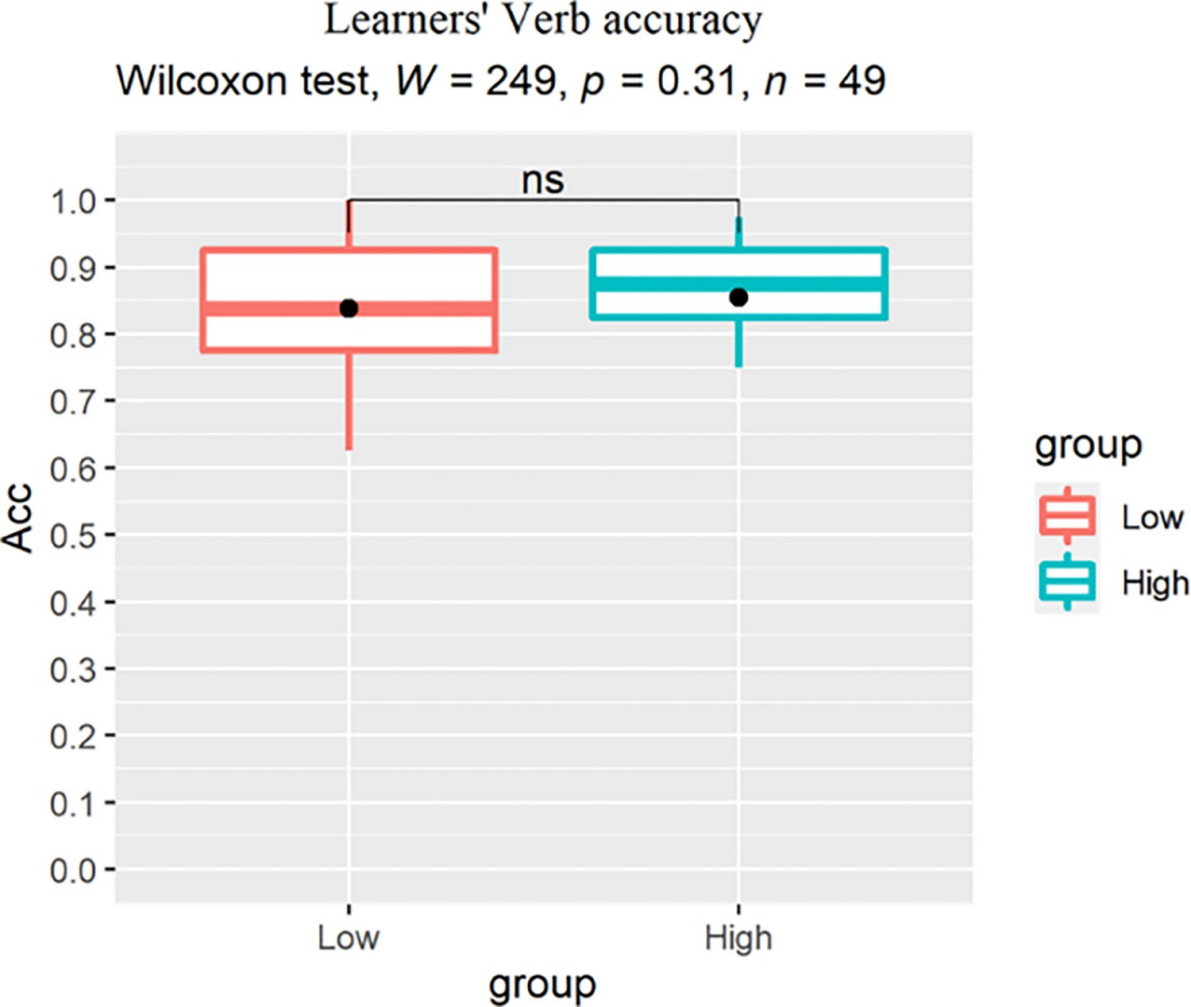
Learners’ accuracy (%) for the Verb structure, dots indicate group-level means.

**Fig 9:**
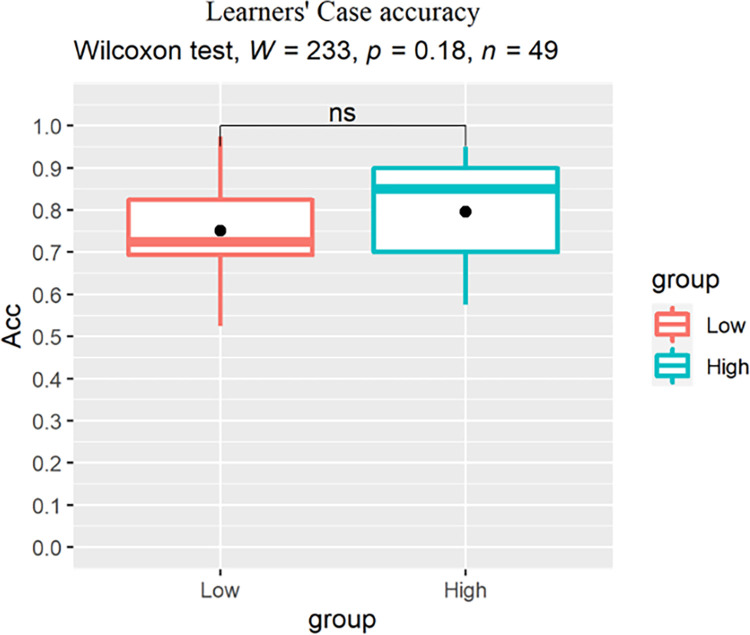
Learners’ accuracy (%) for the Case structure, dots indicate group-level means.

For between-group analysis, we conducted a Wilcoxon rank-sum test for each condition to reveal the main effect of proficiency on learners’ judgement accuracy. For the ADJ condition, a significant effect of group was found (p = 0.0063, r = 0.392) with the low proficiency learners scoring lower than their high proficiency peers (Fig 7). For the Verb (Fig 8) and Case conditions (Fig 9), no significant group difference was revealed (Verb: p = 0.31, r = 0.146; Case: p = 0.18, r = 0.192), indicating that increase in proficiency did not result in enhanced explicit knowledge of the L2-shared or L3-unique structures.

To reveal the interaction between condition and proficiency, we fitted the judgement data into a mixed-effect logistic regression model with the glmer function in R. In addition to the high-proficiency group’s overall higher accuracy, the model estimated a significant interaction effect (F = 4.4823, p<0.001). Specifically, an increase in proficiency further consolidated the learners’ advantage in explicit knowledge of the ADJ condition over that of Verb (p = 0.008) and Case (p = 0.006).

#### Self-paced reading reaction time

**Fig 10:**
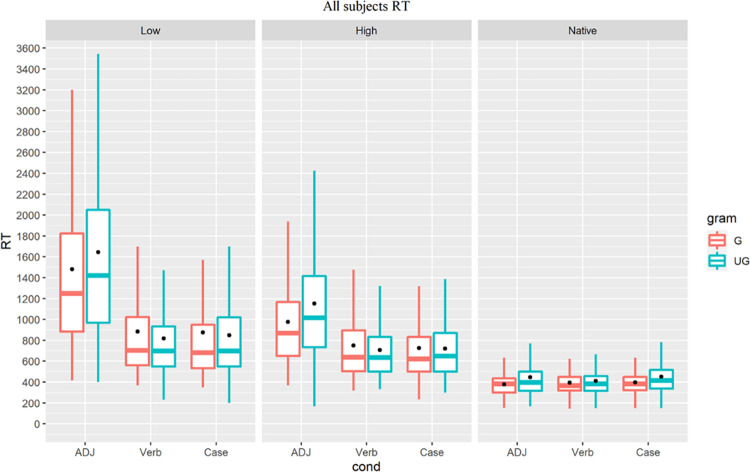
All groups’ RT, colored for grammaticality.

The overall RT distribution is shown in Fig 10. Analyses were implemented in R using the linear mixed effect model (LMMs, the lmer() function in R) fit by restricted maximum likelihood (REML) with grammaticality as the fixed effect and participant and trial item as random effects. For each group, we fitted the data into a mixed model to estimate the fixed effect of grammaticality with random intercepts of individual and item, which resulted in three LMMs for the three target conditions, along with a reduced model without the fixed effect for each LMM to reveal any robust difference in model fit.

**Fig 11:**
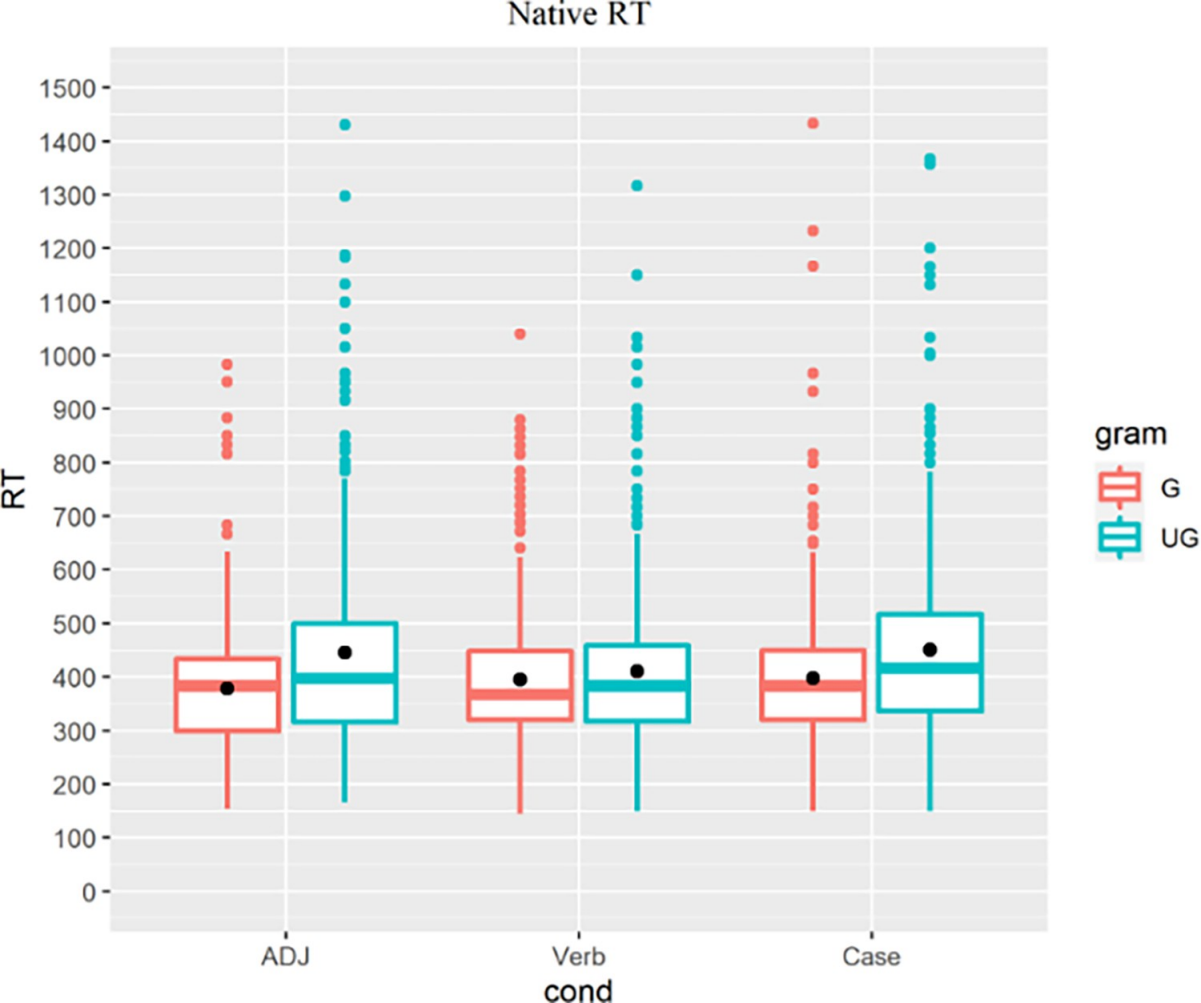
Native speakers’ RT, colored for grammaticality.

The native controls’ RT (Fig 11) showed an effect of grammaticality in all three conditions (ADJ: t = 6.991, p<0.001; Verb: t = 2.126, p = 0.034; Case: t = 6.476, p<0.001), confirming the validity of the experimental stimuli.

**Fig 12:**
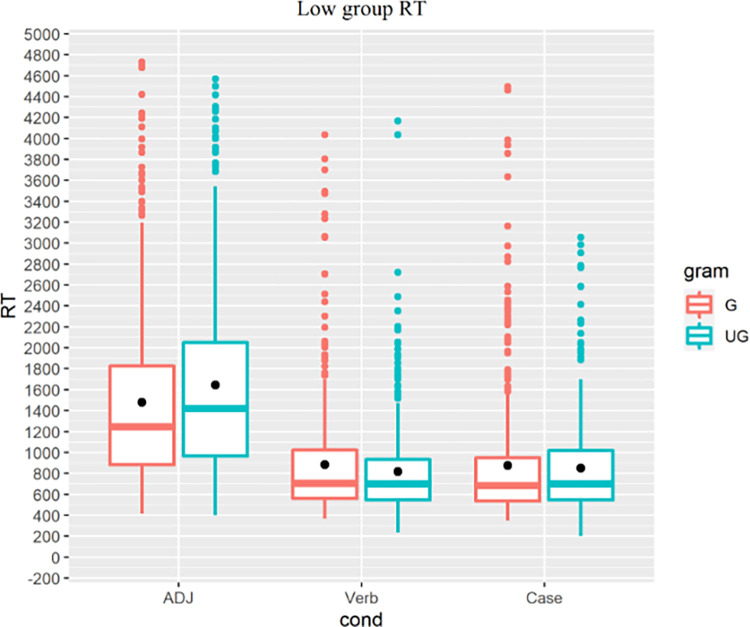
Low proficiency group’s RT, colored for grammaticality.

For the low proficiency group (Fig 12), LMMs returned a main effect of grammaticality for the L1-shared ADJ condition (t = 3.792, p<0.001) but not the Verb (t = -2.535, p = 0.011) or Case condition (t = -0.936, p = 0.35). These RT results indicate that despite their limited experience with the Korean language, low proficiency learners had developed implicit knowledge of the Korean prenominal adjective structure that shares similarity with L1-Cantonese.

**Fig 13:**
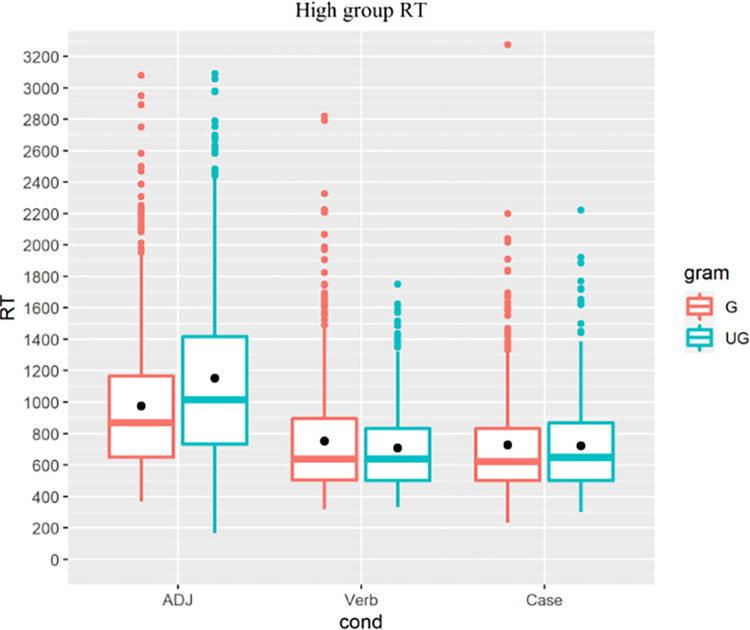
High proficiency group’s RT, colored for grammaticality.

For the high proficiency group (Fig 13), similarly, LMMs returned a main effect of grammaticality for the L1-shared ADJ condition (t = 6.567, p<0.001) but not the Verb (t = -2.558, p = 0.011) or Case condition (t = -0.345, p = 0.730). Like their low proficiency peers, high proficiency learners exhibited implicit knowledge of the ADJ feature but not of the Verb or Case structure.

Follow-up analysis of the linear mixed models was conducted with the R package simr to reflect the power of our observed effect of grammaticality on learners’ implicit processing of the prenominal adjectival marker. The power for the predictor of grammaticality was 97% for the low proficiency group (with a 95% confidence interval of 95.74% to 97.97%) and 100% for the high proficiency group (with a 95% confidence interval of 99.63% to 100%).

To further explore if learners’ implicit knowledge of the adjective marker was influenced by proficiency, we built another LMM with fixed effects of grammaticality, proficiency and the interaction between them, with random intercepts of participants and trial items, to reveal if an increase in proficiency would result in implicit knowledge enhancement. The model returned main effects of grammaticality (t = 5.110, p<0.001) with the ungrammatical trials requiring longer RT than the grammatical trials, and proficiency (t = 4.115, p<0.001) with the higher proficiency learners showing overall faster RT than the lower proficiency learners, but no grammaticality-proficiency interaction (t = -0.394, p = 0.694).

Lastly, a three-way interaction model was built to reveal the main effect of proficiency, structural condition and grammaticality and their interactions on the learners’ reading time of the critical word. Proficiency showed no influence on the interaction between structural condition and grammaticality (F = 0.0005, p = 0.999).

### Discussion

Results from the two tasks suggested that similarity-driven CLI from both L1 and L2 had a facilitative effect on the acquisition of L3 but their facilitations were instantiated in distinct ways. In terms of explicit knowledge, both L1 and L2 had a facilitative influence on the acquisition of L3 induced by L1-L3 or L2-L3 similarity. However, whereas L1-sourced CLI showed continuing facilitation of the development of L3 metalinguistic knowledge which eventually led to native-like (91% accuracy) L3 processing, the effect of L2-origin CLI reached a plateau before the learner achieved near-native metalinguistic awareness of the L2-consistent feature (high: 86%; low: 84%; native: 97%). In addition, high proficiency learners of Korean exhibited similar, plateaued judgement scores for both the Case and Verb structures, despite a head-start for the Verb condition at the early stage of acquisition. Based on such findings, we expect that L2-similarity may expedite learners’ metalinguistic awareness of L3 morphosyntax but has no modulating effect on ultimate L3 attainment as we did not observe a proficiency-induced change in L2-similarity facilitation outcomes. Implicit L3 knowledge, on the other hand, only benefitted from L1-sourced CLI induced by L1-L3 similarity. Furthermore, since implicit knowledge of the L1-consistent Korean structure was observed at low proficiency levels, we speculate that similarity-induced proceduralization takes place early in learners’ contact with the L3. However, the implicit proficiency appeared to stabilize at a certain level, as attested by the consistent SPRT performance across groups.

Our findings of differential facilitation effects of L1 and L2 on implicit and explicit L3 knowledge are consistent with the predictions of the D/P model that posited distinct functions of the procedural and declarative memory systems in subserving language [2]: L1 morphosyntactic knowledge stored in the procedural memory system has a consolidating effect on learners’ proceduralization of L3 grammar, whereas L2 morphosyntactic knowledge, especially that acquired from classroom instructions, is primarily sustained by declarative memory and assumed to facilitate explicit L3 processing. Contrary to the prediction made by the L2 Status Factor [13], our findings verified the transferability of L1 implicit knowledge. Previously mentioned SLA studies that found supporting evidence of L1-similarity-induced enhancement on implicit L2 knowledge further call the presumed impermeability of memory systems into question [26,27].

## Experiment 2: EEG experiment

The EEG experiment aimed at revealing the neurocognitive underpinnings of L1 and L2 crosslinguistic influence (CLI) on L3 processing by scrutinizing learners’ neural signatures during the processing of L1-shared, L2-shared and L3-unique structures. Focusing on the early stage of L3 acquisition^2^, we examined low proficiency L3 learners’ brain responses under L1 and L2 facilitation. Building on previous findings of L2/L3 learners’ native-like neural processing of the L1-shared morphosyntactic feature and our behavioral findings on L3 learners’ implicit knowledge of the L1-shared feature, we predicted that similarity-driven L1 CLI would lead to a native-like P600 brain response for the L1-shared structure in our L3 learners. On the other hand, based on the possible correlation between the meaning-oriented N400 component and the recruitment of the declarative memory proposed in Ullman’s recent discussion of the D/P model [2], we predicted that L2-L3 similarity would give rise to the N400 response during L3 structural analysis. A total of twelve L3 learners’ electrophysiological data was collected and is presented as preliminary data in this section.

### Methodology

#### Participants

Twenty-four participants participated in the EEG experiment, including 12 low-proficiency Korean learners and 12 Korean native speakers born and raised in Korea.

#### Experimental materials

The EEG experiment tested the same three structures as the web-based behavioral experiment, namely the prenominal adjectival marker (ADJ), simple past-tense inflection (Verb) and accusative case marking (Case) structures. The experimental stimulus sentences were the same ones generated for the web-based experiment (Table 1).

#### Procedure

**Fig 14:**
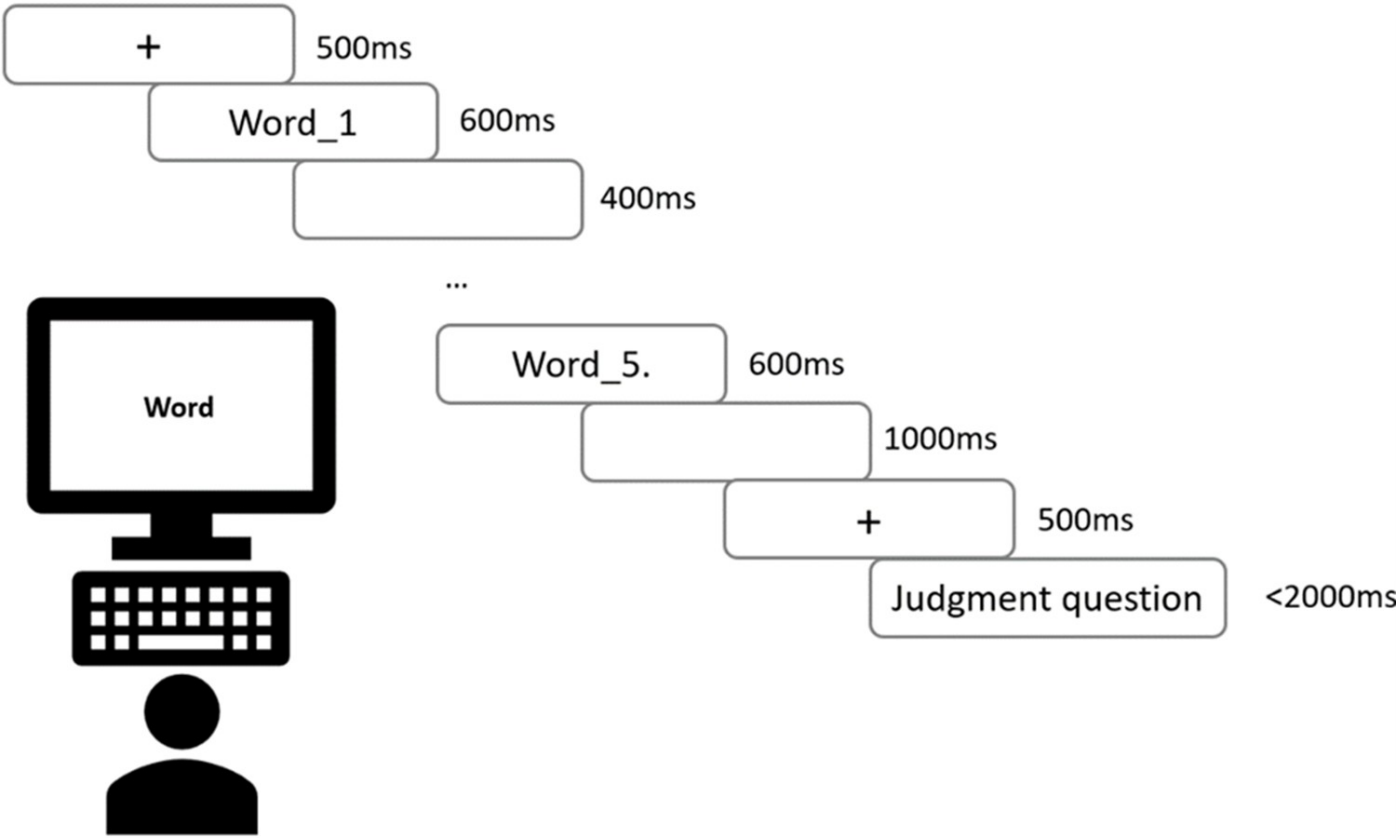
Trial demonstration of the EEG grammaticality judgement task.

Participants were seated on a chair roughly 1 meter in front of the monitor and were instructed to read each Korean sentence on the screen and make a grammaticality judgement as quickly as possible by pressing a pre-assigned response key. Sentences were presented in black letters with a silver background using E-prime (Version 2.0, Psychology Software Tools, Pittsburgh, PA). Fig 14 shows a trial demonstration of the EEG task. The word presentation duration in the current study was 600ms, which is different from the 500ms duration that is typical adopted in EEG studies with Indo-European languages, in order to accommodate the longer time needed for reading Korean words. Korean language has a writing system that is more complex than both traditional Chinese and English, as it is an alphabetic language that organizes letters into blocks to form characters. A Korean character is formed by at least 2 and at most 4 letters, and the longest word in the experiment stimuli had up to 4 characters, which means a total of 16 letters in a single word. A longer word length on top of an alphabetic block structure makes Korean more challenging to read. In addition, pilot learner subjects were tested and interviewed about the stimulus presentation before the experiment paradigm was finalized, and the outcome suggested a minimum presentation duration of 600ms. Therefore, the current experiment adopted the 600ms duration for word presentation.

A total of 340 experimental trials were presented in randomized order. Four lists were created to counterbalance response hands and stimulus grammaticality. The entire task was divided into 4 blocks, each containing 85 trials. In the middle of each block and in between blocks, participants were instructed to take a short break.

#### EEG recording

**Fig 15:**
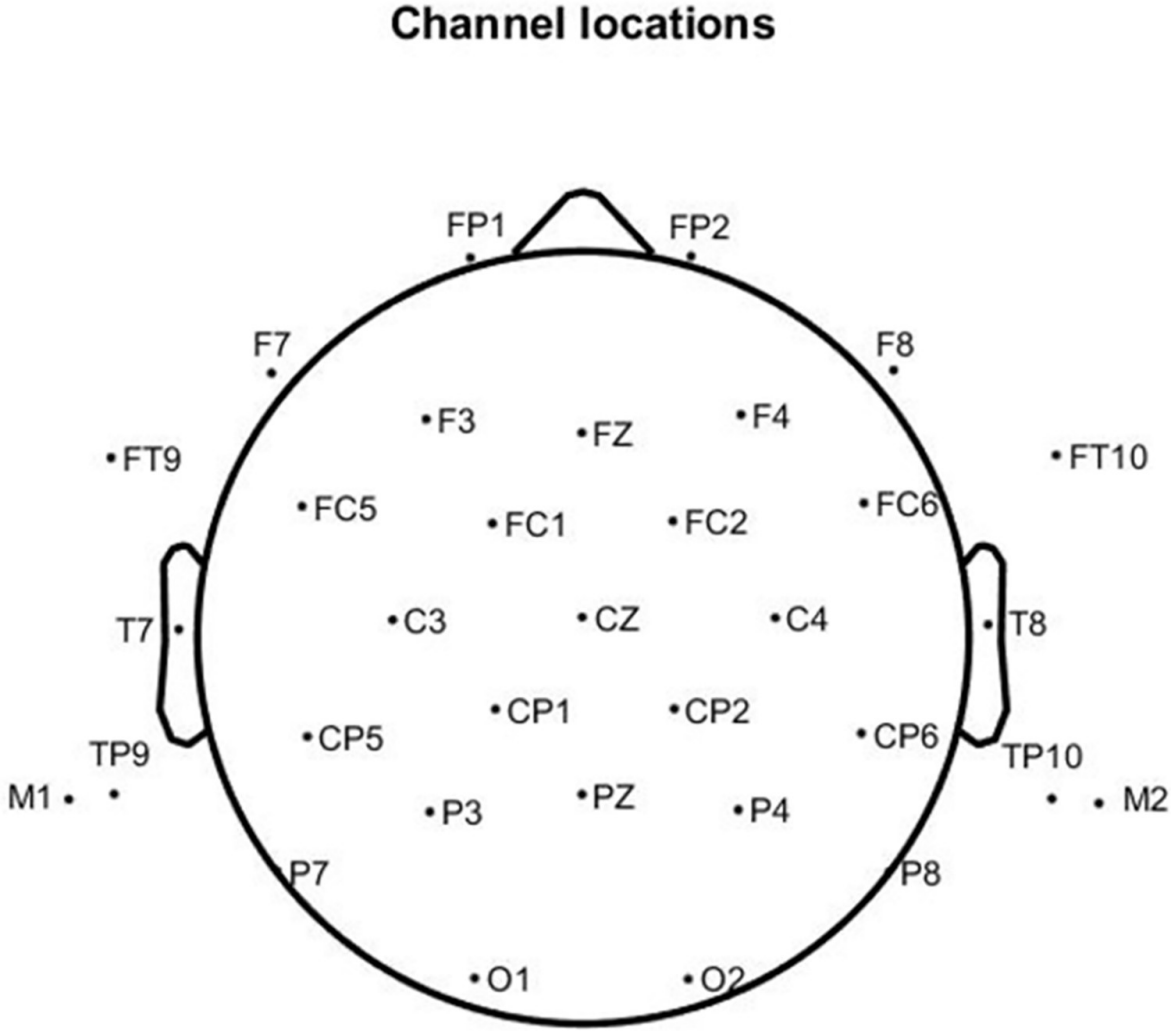
Channel location map.

Continuous EEG data were recorded with the Neuroscan system from 31 active Ag/AgCl sintered electrodes attached to an electrode cap as well as from electrodes placed on the left and right mastoids (Fig 15). EEG was referenced online to the CPZ. Eye movements were recorded from two bipolar channels with electrodes placed on the left and right temples (i.e., the HEOG) and above and below the left eye (i.e., the VEOG). The EEG was amplified with a Synamps RT amplifier, digitized with a 500-Hz sampling rate. Impedance for all EEG electrodes was kept below 5 kΩ.

#### EEG preprocessing

Offline preprocessing was done with the EEGLAB toolbox (Delorme & Makeig, 2004) in MATLAB (R2019a; MathWorks). EEG data were re-referenced offline to the average activity over the left and right mastoids, down-sampled to 250 Hz, and band-pass filtered for 0.5~30 Hz. Data segments with artifacts were auto-rejected with the built-in function of EEGLAB. The continuous EEG data were then cut into condition-specific -200 millisecond (ms) to 1000ms epochs for the native Korean participants and -200ms to 1200ms epochs for the learners, with baseline correction to the average EEG activity in the pre-stimulus 200-ms interval. Heartbeats, eye movements and muscle movements were removed via independent component analysis (ICA). Trials with amplitudes below -75 μV or above 75 μV were rejected from statistical analysis.

#### EEG analysis

**Fig 16:**
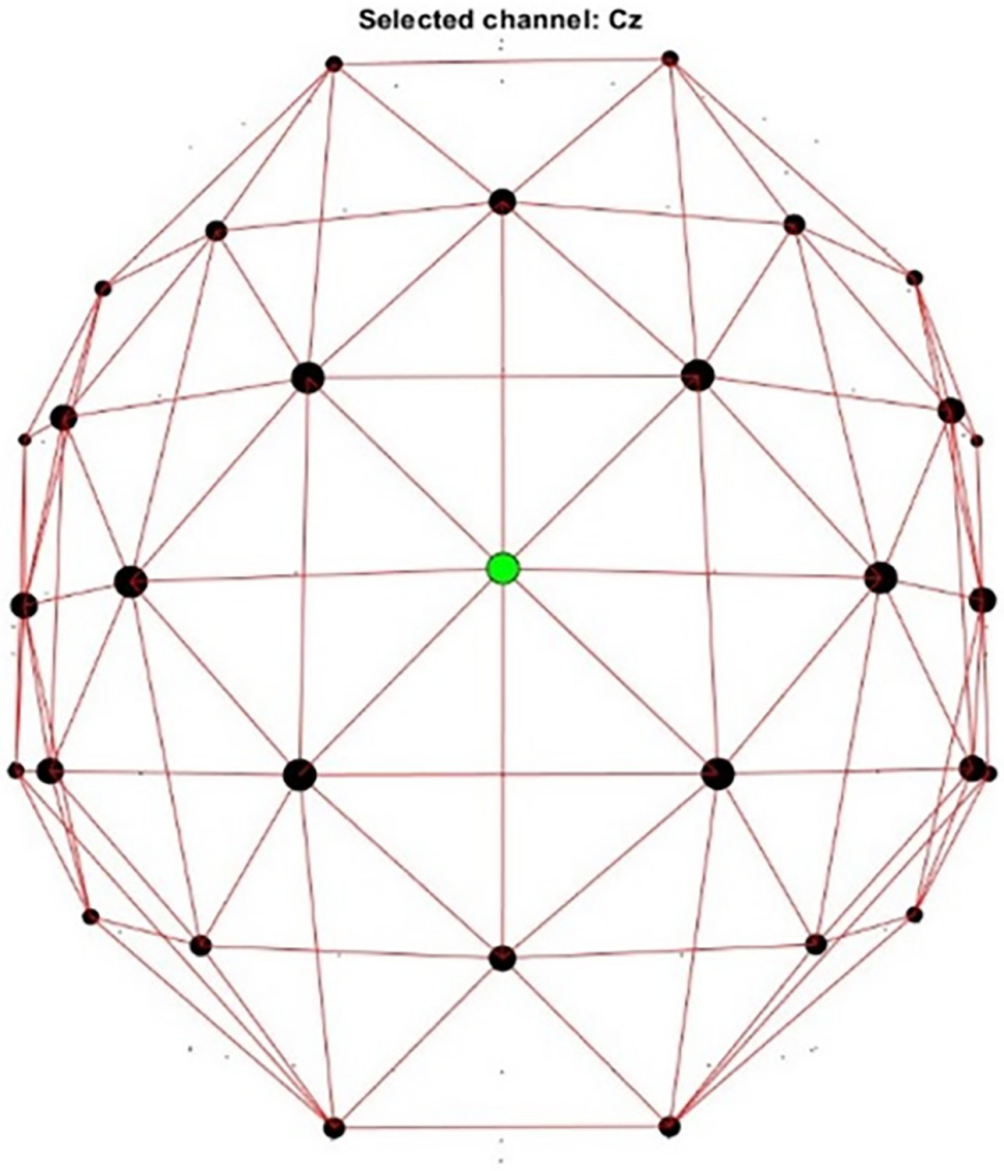
Neighbour plot for the cluster-based permutation analysis (Cz highlighted).

EEG data were analyzed using nonparametric cluster-based permutation tests with 2000 iterations (α_cluster_ = 0.05). A cluster encompassed a minimum of 4 neighbouring channels (3 for a cluster located at the edge of the scalp, Fig 16). Since syntactic ERP components in language learners vary a lot in comparison to native speakers, traditional ERP analysis based on the components’ polarity and amplitude within a pre-determined time window seemed inappropriate, especially given the relatively small sample size. By repeating randomization tests on the waveforms, the nonparametric cluster-based permutation tests allow the construction of probability distribution and reveal any effect of grammaticality on the morphosyntactic processing. In addition, nonparametric cluster-based permutation requires fewer pre-determined vectors and corrects for multiple comparisons to minimize Type 1 errors. Within-group permutation tests were conducted on pairwise differences between the grammatical and ungrammatical conditions. ERPs were measured for the critical word of each condition (Word_3 and Word_4 for ADJ, Word_5 for Verb and Word_4 for Case, see Table 5). Note that for the ADJ condition, Word_3 was the earliest region for grammaticality judgement. However, the prenominal adjectival marker is attached locally to the adjective in Korean but is not necessarily bound to the adjective in Chinese. In Chinese languages, morphological inflection is largely lacking with each character being a morpheme and spacing between morphemes that together form a word and/or between words that form a sentence does not affect grammaticality. For examples in Cantonese (Table 5), 1) has the prenominal marker attached to the adjective, 2) has it attached to the noun, and 3) leaves it unbound, and all three analyses are theoretically possible. This may lead to Cantonese-speaking Korean learners relying on the complete noun phrase (adj. + noun-head) for making morphosyntactic judgments. Consequently, sensitivity towards the prenominal adjectival marker could be triggered at either the adjective or the noun head, so we tagged both Word_3 and Word_4 for the ADJ condition.

**Table 5:** Example sentences for each condition.

Position:	Word_1	Word_2	Word_3	Word_4	Word_5
**ADJ condition (L1-L3 congruent)**					
Grammatical:	Jaemin-TOP	home-LOC	spicy-**PreN**	bibimbap-ACC	eat.
Ungrammatical:	Jaemin-TOP	home-LOC	spicy-**PostN**	bibimbap-ACC	eat.
**Verb condition (L2-L3 congruent)**					
Grammatical:	Annie-TOP	yesterday	after dinner-LOC	movie-ACC	watch**ed**.
Ungrammatical:	Annie-TOP	yesterday	after dinner-LOC	movie-ACC	watch.
**Case condition (L3-unique)**					
Grammatical:	Martin-TOP	morning-LOC	tennis-**ACC**	often	play.
Ungrammatical:	Martin-TOP	morning-LOC	tennis-**NOM**	often	play.

### Results

#### Behavioral results

The accuracy score of the timed GJT was calculated as the ratio of correctly judged sentences over all sentences responded to (Table 6). Trials without a response were rejected.

**Table 6:** Group-level means and SDs for timed GJT accuracy.

	ADJ	Verb	Case
**Low**	0.83 (SD = 0.14)	0.78 (SD = 0.10)	0.66 (SD = 0.11)
**Native**	0.96 (SD = 0.03)	0.96 (SD = 0.03)	0.94 (SD = 0.04)

**Fig 17:**
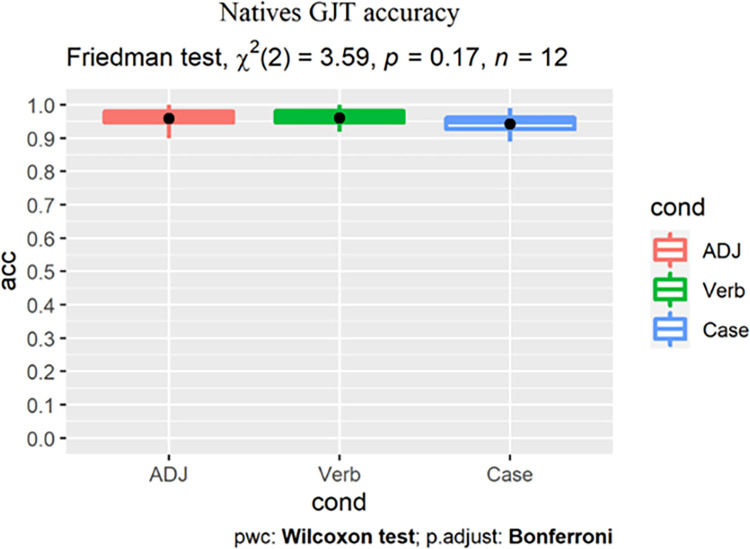
Native Korean speakers’ timed GJT accuracy (%) in the three conditions.

Friedman tests were conducted within a participant group to reveal the effect of structure, followed by post-hoc pairwise Wilcoxon signed-rank tests with Bonferroni correction to correct for multiple comparisons. Statistical analysis was performed in R (Version 4.1.0, R Core Team, 2021). For the native group, Friedman test revealed no effect of condition (χ^2^(2) = 3.59, p = 0.17), with the post-hoc pairwise comparisons returning no difference between any two conditions (Fig 17).

**Fig 18:**
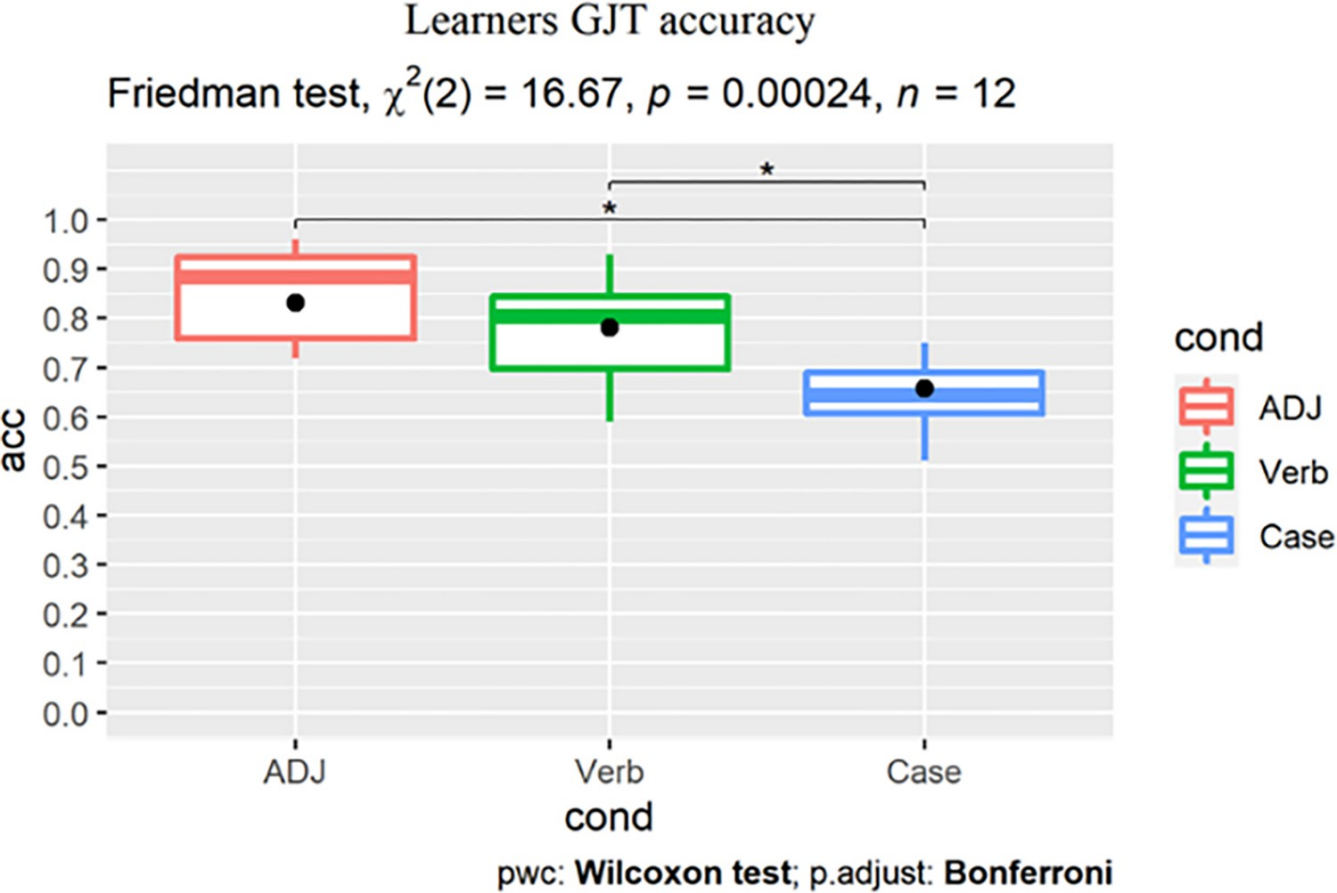
Learners’ timed GJT accuracy (%) in the three conditions.

For the learner group, Friedman test revealed a main effect of condition (χ^2^(2) = 16.67, p<0.001), with significantly higher scores in the ADJ (adjusted p = 0.016, r = 0.816) and Verb conditions (adjusted p = 0.032, r = 0.748) than Case. Learners’ accuracy scores for the ADJ and Verb conditions showed no robust difference (adjusted p = 0.113, r = 0.612, Fig 18).

#### EEG results

EEG statistical analysis was based on all trials, whether or not participants judged the grammaticality correctly, to avoid response bias [56]. The statistical report focuses on the time windows for the ELAN, LAN, N400 and P600 components.

**Fig 19:**
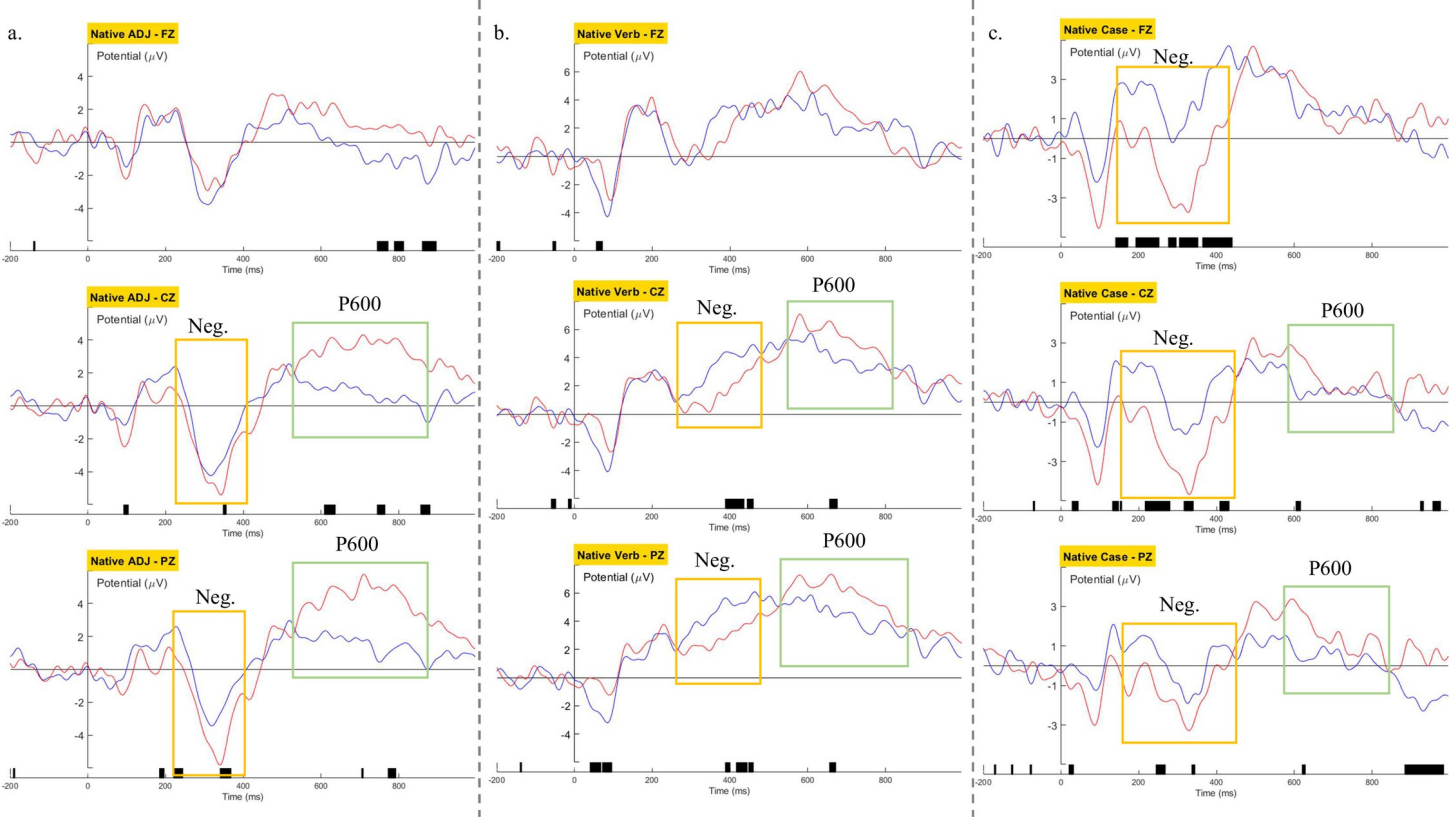
Native Koreans’ grand averaged waveforms on Fz, Cz and Pz regions. (a) the ADJ condition. (b) the Verb condition. (c) the Case condition. Blue: Grammatical; Red: Violation. Bars indicate time windows with a significant effect of grammaticality, with marked early negativity (yellow box) and P600 (green box) components.

For the native group, cluster-based permutation tests revealed a main effect of grammaticality for the negative clusters within the typical N400 time window and the positive clusters within the typical P600 time window. For the ADJ condition, a negative cluster was found over the centroparietal locations (central: 372-385ms, p = 0.07; posterior: 332-364ms, p = 0.008) and a widespread positivity was observed (central: 612-884ms, p = 0.027; posterior 696-820ms, p = 0.026; Fig 19A). For the Verb condition, a negative cluster (central: 384-428ms, p = 0.016; posterior: 388-452ms, p = 0.031) and a positive cluster spread over the centroparietal channels (central: 632-676ms, p = 0.048; posterior: 656-680ms, p = 0.047; Fig 19B). For the Case condition, a widespread negativity (frontal: 340-456ms, p = 0.006; central: 372-432ms, p = 0.016; posterior: 364-370ms, p = 0.035) and a centroparietal positivity were observed (central: 602-660ms, p = 0.047; 604-628ms over the posterior electrode, p = 0.041; Fig 19C).

Overall, native Koreans’ brainwaves showed the typical morphosyntactic processing pattern of early negativity followed by centroparietal positivity. Although the distribution of the observed negativity did not coincide perfectly with previously reported LAN component, our Korean natives displayed a brainwave pattern largely comparable to their Indo-European language-speaking peers.

**Fig 20:**
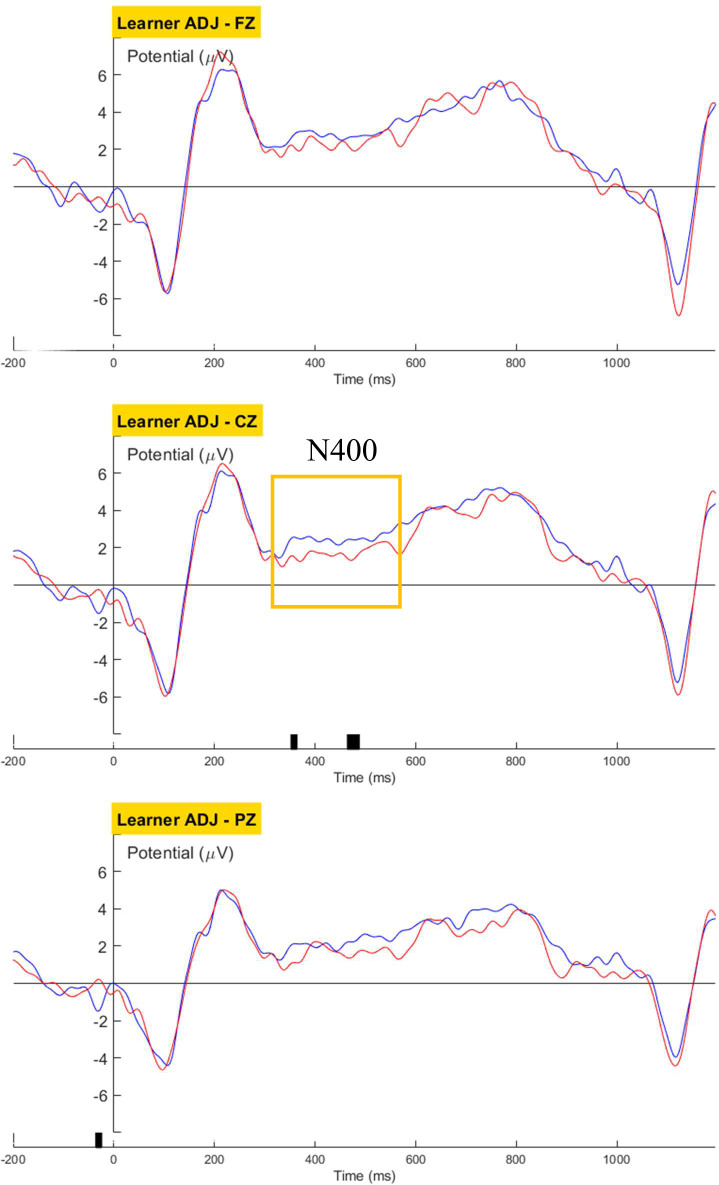
Learners’ grand averaged waveforms for Word_3 of the ADJ condition.

**Fig 21:**
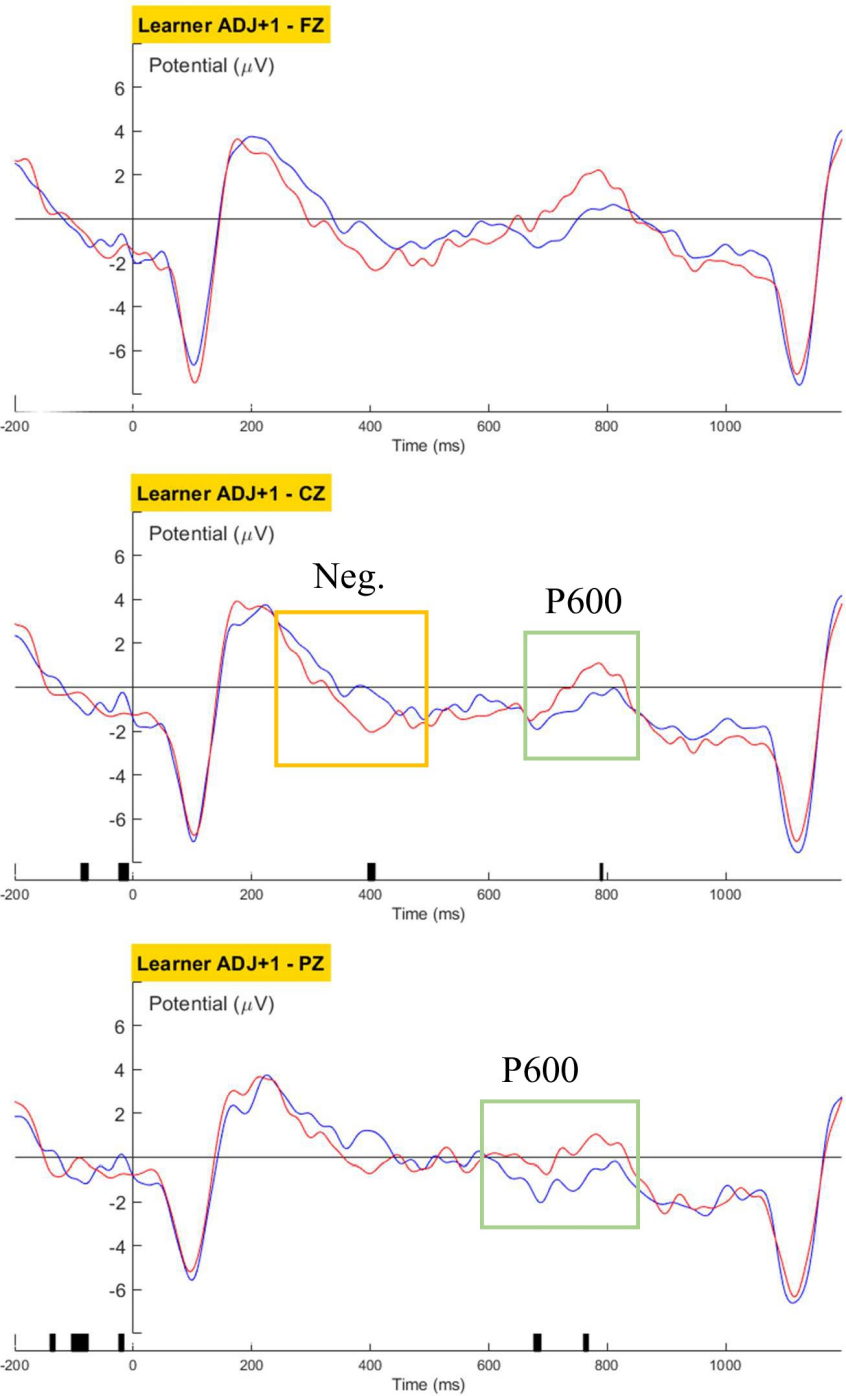
Learners’ grand averaged waveforms for Word_4 of the ADJ condition.

Group-level grand-averaged waveforms for the learners were generated and cluster-based permutation tests were carried out to reveal significant main effects of grammaticality for the three structures. For the ADJ condition, a negative deflection was found over the central channel (356-480ms, p = 0.04; Fig 20), possibly an attenuated N400 induced by the malformed adjective. For the ADJ+1 condition, with the 4^th^ word being the region of interest, learners’ ERP displayed a negative cluster over the central channel (384-408ms, p = 0.01) followed by a centroparietal positivity (central: 776-792ms, p = 0.04; posterior: 680-784ms, p = 0.045; Fig 21). Lateralization of the grammaticality effect was not observed. A LAN or ELAN component was not observed in the learners’ neural response. The learners’ observed brain response of a typical negativity-to-positivity continuum for the noun-head region, despite the delayed latency, was in accordance with native-like brain responses to the morphosyntactic violation. The results also indicate that, although the ungrammatical adjective form possibly elicited a sense of awkwardness or rarity at the position of the target word (i.e., the adjective), the learners did not decompose the adjectival marker syntactically until the end of the noun phrase (i.e., at the noun head). Examples in Table 7 demonstrate the flexibility of the position of the prenominal adjectival marker in Cantonese, and it was expected that such flexibility could influence how Cantonese-speaking learners process adjective-noun phrases in Korean. The learners’ P600-like response at the noun head corroborated such expectation. As native speakers of a language that lacks inflectional morphology, the learners showed reliance on the complete noun phrase to process the adjectival marker.

**Table 7:** Example adjective-noun phrases in Cantonese.

1)	hou2mei6**ge3** ping4gwo2
2)	hou2mei6 **ge3**ping4gwo2
3)	hou2mei6 **ge3** ping4gwo2
	delicious apple

**Fig 22:**
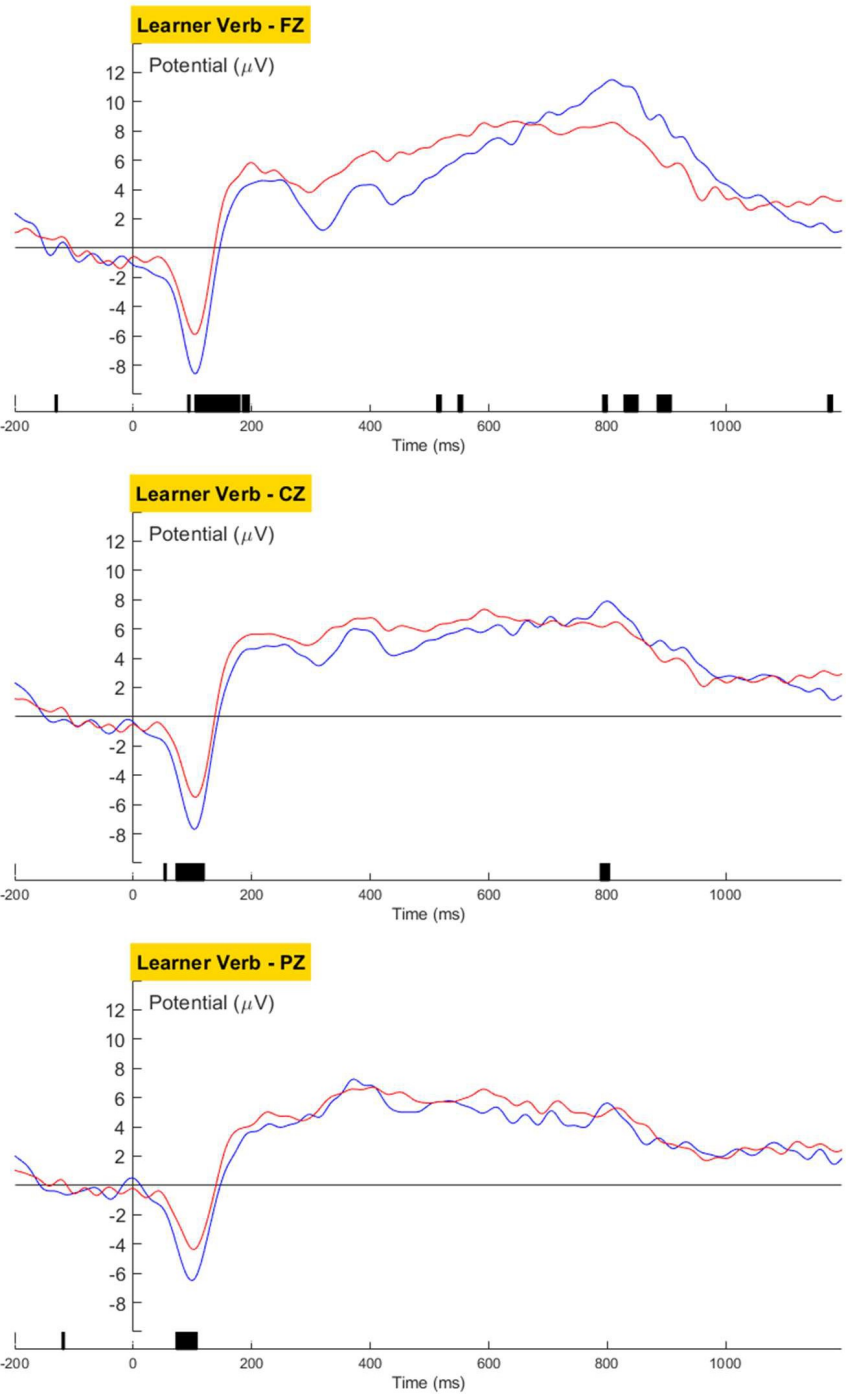
Learners’ grand averaged waveforms for the Verb condition.

**Fig 23:**
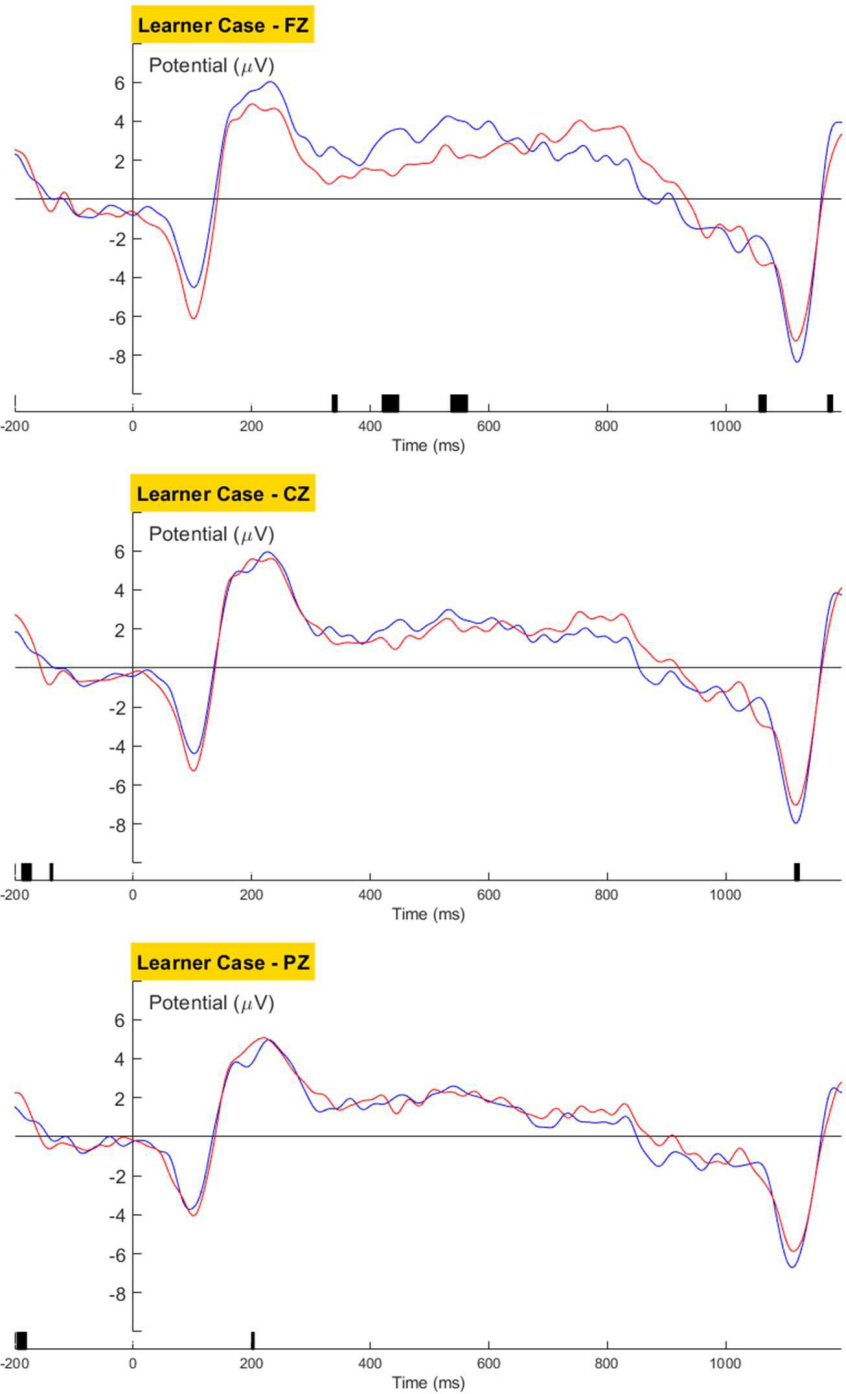
Learners’ grand averaged waveforms for the Case condition.

No language-related ERP components was observed in the learners’ brain response towards violations in the Verb (Fig 22) or Case condition (Fig 23).

### Discussion

Behaviorally, the L1-shared prenominal adjectival structure evoked the highest judgement accuracy score in L3 learners, followed by the L2-shared verb inflection which showed better performance than the L3-unique case marking. The early-stage learners’ grammaticality judgement accuracy in the EEG experiment coincided with that of the low proficiency group in our web-based behavioral experiment, lending further support to the facilitative influence of both L1-L3 and L2-L3 similarity on the consolidation of L3 metalinguistic knowledge.

ERP analysis revealed that violation in the L1-shared ADJ structure evoked a biphasic negativity-P600 pattern, a processing marker typically presented in native speakers’ morphosyntactic repair/re-analysis, in our L3 early-stage learners, suggesting that overlap between L1 and L3 played an important role in expediting L3 proceduralization. Earlier studies that examined L2 learners’ morphosyntactic processing have reported similar facilitation of L1-L2 overlap on the internalization of L2 grammar (for a review, see [45]). However, as Chinese languages generally lack inflectional morphemes and treat each character as a morpheme, we predicted that the L1-Cantonese learners of Korean would reserve their grammatical judgement of the adjectival form until the presentation of its paired noun-head. The ERP results confirmed our prediction as the native-like brain response towards adjectival structure violation was elicited upon the appearance of the noun head instead of the adjective.

Morphosyntactic violation elicited no observable language-related neural response for the Verb or Case condition in L3 Korean learners. Despite learners’ better behavioral performance for the L2-shared verb inflection than the L3-unique case marking, no neurological indication of processing differences was observed. This may be because L2-L3 similarity, despite facilitating explicit grammatical knowledge, does not contribute to the proceduralization of L3 grammar. Taken together with learners’ native-like processing of the ADJ structure, the null brain response towards the L2-shared verb inflection suggests that lack of L1-similarity is the primary reason for the commonly observed difficulty in morphological processing for speakers whose L1 lacks inflectional morphology.

We acknowledge that the sample size of the EEG study is relatively small for the purpose of drawing conclusions about the crosslinguistic facilitation of L3 acquisition, and our preliminary analysis of the learners’ neural responses aims to provide exploratory findings on the effects of crosslinguistic similarity on subsequent language acquisition. Future research studies with larger sample sizes are needed to reach a deeper and more comprehensive understanding of such effects.

## General discussion

Overall, the results of the behavioral task and the EEG experiment provided compelling evidence for facilitative crosslinguistic influence of both L1 and L2 on L3 morphosyntactic acquisition when inter-language similarity is present. However, L1 and L2 were found to influence performance in L3 morphosyntax differently. To account for these findings, we suggest that the L1 and L2 differentially facilitated implicit and explicit processing of L3: the similarity-driven crosslinguistic influence from L1 showed a consolidating effect on the implicit knowledge and proceduralization of L3 grammar, whereas the L2 facilitation merely elicited an enhancement of L3 metalinguistic knowledge that led to better performance in explicit structural analysis but showed no modulating effect on learners’ online L3 processing.

This interpretation of our findings is concordant with the hypotheses of the D/P model [2] that predicted distinct involvement of the declarative and procedural memory systems in the acquisition and sustaining of native and non-native languages. In accordance with the predominant recruitment of procedural memory in L1 sustainment, we found strengthened L3 procedural knowledge of the morphosyntactic feature shared by L1 and L3. Consistent with the engagement of declarative memory in L2 morphosyntactic processing as predicted under the D/P model, L3 learners exhibited enhanced explicit knowledge of the L2-similar structure. Additionally, by combining the behavioral findings with the EEG outcomes, we found that L1-similarity led to both enhanced procedural L3 knowledge and native-like L3 processing as manifested in the negativity-P600 biphasic brain response, suggesting an association between the recruitment of procedural memory and the ERP marker (i.e., P600) for native-like processing. Although Ullman [2] discussed the correlation between the involvement of declarative memory and the N400 ERP response, our early-stage L3 learners did not show any robust violation-induced brain reaction during online processing of the L2-shared feature. One possibility is that our learners’ proficiency in Korean was too low to show observable online sensitivity to violations of the L2-shared feature.

There has been debate over how individual differences in procedural and declarative memory abilities contribute to varying language learning outcomes. Past findings revealed that procedural memory ability is a statistically significant predictor of better learning outcomes for adult language learners [57]. However, the predictive power of memory abilities on L2 learning outcomes was only observed for learners in an implicit learning context. Considering that the L3 learners in the present study learned Korean explicitly in a classroom setting, we believe that individual differences in procedural or declarative memory abilities had limited influence on the observed results.

As many previous studies on crosslinguistic influence compared the same learner group’s performance on different structures [27,42,58,59], one critical factor that has caused concern is the intrinsic difficulty of one structure over another. Morphosyntactic features may differ in structural complexity, required level of cognitive resource, and learnability, rendering some structures harder than others. In L3 acquisition, researchers have tried to work around structural differences by testing mirror-group learners. Such designs are not without limitations as the two learner groups’ L2 proficiency is difficult to measure and render comparable. In our study, we cannot rule out the possibility that in terms of structural complexity and learnability, the adjectival marker is the easiest, followed by the verb inflection and then case marking, which could lead to the same results. However, our learners were taught the three structures in the order of case marking first, then the simple past-tense and lastly the prenominal adjectival form, and hence learners were expected to be most experienced with the case-marking system and least familiar with the adjectival structure. The results revealed the exact opposite pattern as learners performed best on the adjective structure, followed by verb inflection and then case. Moreover, a previous study on the acquisition of Korean case marking by L1-Japanese and L1-English speakers found that Japanese-speaking learners benefited from L1-L2 similarity in the case marking system and robustly outperformed their L1-English peers [60]. Another study showed an enhancing effect of L2-Japanese on L3 Korean acquisition of case marking, adding further support to similarity-driven facilitation [61]. Therefore, we believe that our observed acquisition patterns resulted from the facilitative effect of L1-L3 and L2-L3 similarity rather than from structural differences.

We acknowledge the relatively small sample size of the EEG experiment, which is one of the limitations of investigating natural language learners, particularly under pandemic conditions. Another limitation of the current study is the lack of L2 proficiency measures. While the language demographics of HK local students were discussed in the participant description in the Method section, the findings could be strengthened by incorporating objective measures of the learners’ L2 proficiency, and we encourage future research to include measurements of L2 proficiency for a more comprehensive understanding of L2 influences. The results of this study should therefore be considered preliminary. Nonetheless, we believe our EEG findings provide valuable insights into the neurocognitive processing of L3 morphosyntax and the neural mechanisms of CLI, helping to build a foundation for further neuroimaging studies on CLI.

## Supporting information

S1 Checklist (DOCX)
